# Relationship of tobacco smoking to cause-specific mortality: contemporary estimates from Australia

**DOI:** 10.1186/s12916-025-03883-9

**Published:** 2025-02-25

**Authors:** Grace Joshy, Kay Soga, Katherine A. Thurber, Sam Egger, Marianne F. Weber, Peter Sarich, Jennifer Welsh, Rosemary J. Korda, Amelia Yazidjoglou, Mai T H Nguyen, Ellie Paige, Michelle Gourley, Karen Canfell, Emily Banks

**Affiliations:** 1 National Centre for Epidemiology and Population Health (NCEPH), The Australian National University, Acton, Canberra, ACT 2601 Australia; 2 The Daffodil Centre, The University of Sydney, A Joint Venture With Cancer Council NSW, Sydney, NSW Australia; 3 Population Health Program, QIMR Berghofer Medical Research Institute, Brisbane, Australia; 4 School of Public Health, University of Queensland, Brisbane, Australia; 5 Burden of Disease and Mortality Unit, Australian Institute of Health and Welfare, Canberra, Australia; 6 Sydney School of Public Health, University of Sydney, Sydney, Australia

**Keywords:** Smoking, Tobacco, Cause of death, Fatal burden of disease

## Abstract

**Background:**

Tobacco industry activities and reduced smoking prevalence can foster under-appreciation of risks and under-investment in tobacco control. Reliable evidence on contemporary smoking impacts, including cause-specific mortality and attributable deaths, remains critical.

**Methods:**

Prospective study of 178,169 cancer- and cardiovascular-disease-free individuals aged ≥ 45 years joining the 45 and Up Study in 2005–2009, with linked questionnaire, hospitalisation, cancer registry and death data to November 2017. Cause-specific mortality hazard ratios (HR) by smoking status, intensity and recency were estimated, adjusted for potential confounding factors. Population attributable fractions were estimated.

**Results:**

There were 13,608 deaths during 9.3 years median follow-up (1.68 M person-years); at baseline, 7.9% of participants currently and 33.6% formerly smoked. Mortality was elevated with current versus never smoking for virtually all causes, including chronic lung disease (HR = 36.32, 95%CI = 26.18–50.40), lung cancer (17.85, 14.38–22.17) and oro-pharyngeal cancers (7.86, 4.11–15.02); lower respiratory infection, peripheral vascular disease, oesophageal cancer, liver cancer and cancer of unknown primary (risk 3–5 times as high); and coronary heart disease, cerebrovascular disease and cancers of urinary tract, pancreas, kidney, stomach and prostate (risk at least two-fold); former versus never-smoking demonstrated similar patterns with attenuated risks. Mortality increased with smoking intensity, remaining appreciable for 1–14 cigarettes/day (e.g. lung cancer HR = 13.00, 95%CI = 9.50–17.80). Excess smoking-related mortality was largely avoided with cessation aged < 45 years. In 2019, 24,285 deaths (one-in-every-six deaths, 15.3%), among Australians aged ≥ 45 years, were attributable to tobacco smoking.

**Conclusions:**

Smoking continues to cause a substantial proportion of deaths in low-prevalence settings, including Australia, highlighting the importance of accelerated tobacco control.

**Supplementary Information:**

The online version contains supplementary material available at 10.1186/s12916-025-03883-9.

## Background

Tobacco smoking is a leading avoidable cause of morbidity and mortality internationally [1]. Studies from multiple countries, including the USA [2, 3], UK [4], Japan [5] and the Asia Pacific region [6], have shown that the risk of premature mortality from a range of causes is increased for individuals who currently smoke, compared to people who have never smoked. Tobacco smoke is a well-established carcinogen, known to cause cancer at numerous sites including lung, oro-pharynx, oesophagus, stomach, colorectum, liver, pancreas, kidney, urinary organs, ovary and bone marrow [7]. Smoking also increases the risk of cardiovascular disease (CVD), respiratory illnesses and a range of other conditions [4].

The risk of death from conditions causally related to smoking and the number of smoking-attributable deaths vary according to historic patterns of smoking within a given population, including in relation to prevalence, age at initiation, smoking intensity and duration, and time since cessation. As countries differ in where they stand in the evolution of the tobacco epidemic, relative risk estimates based on one population at a particular time may not necessarily be applicable to another.

To date, estimates of the number of deaths in Australia attributable to tobacco smoking have been based on application of tobacco-attributable fractions from international studies; local data on attributable fractions have not been available. Estimates of smoking-attributable fractions for Australia range from around 16% in 2010 to 7.25% in 2019, amounting to 12,000 to 23,000 deaths annually (Additional file 1: Table S1) [8–14].

While it is well established that smoking increases mortality, contemporary evidence on the impact of smoking and benefits of quitting on premature cause-specific mortality and the proportion of deaths attributable to smoking in Australia is lacking, nor are there any studies using local data on relative risks, and the global evidence-base continues to evolve (Additional file 2: Literature review) [8, 10, 11, 15–55].

This study aims to quantify, using direct data from the Australian population, the relationship of current and past tobacco smoking to cause-specific mortality, and to estimate the number and proportion of deaths attributable to tobacco smoking. Given declining smoking internationally against a background of historically high smoking prevalence, findings are of national and international relevance, particularly for high-income countries at a similar phase in the tobacco epidemic.

## Methods

### Study population

The Sax Institute’s 45 and Up Study is an Australian cohort study of 267,357 men and women aged 45 years and over, randomly sampled from the general population of New South Wales (NSW), Australia, using the Services Australia Medicare enrolment database. Participants living in regional and remote areas and those aged 80 years or over were oversampled. Individuals joined the study from 2005 to 2009 by completing postal questionnaires and giving informed consent for follow-up through repeated data collection and linkage of their data to routinely collected health databases. The general study methods are described in detail elsewhere; the response rate was 19% [56, 57].

Baseline questionnaire data included information on sociodemographic factors (e.g. education, income, country of birth), health behaviours (e.g. smoking, alcohol intake), height and body weight, medical and surgical history, functional capacity and physical activity. To provide data to allow correction for regression dilution, we used repeat data on smoking status from Wave 2 (2012–2015) supplemented by that from the Social, Environmental and Economic Factors sub-study (2010) [56]. Further information regarding the study questionnaires is available at https://www.saxinstitute.org.au/solutions/45-and-up-study/use-the-45-and-up-study/data-and-technical-information/.

Questionnaire data from study participants were linked probabilistically to administrative datasets including fact of death data from the NSW Registry of Births, Deaths and Marriages [58] (1-Jan-2006 to 31-Mar-2019), the Australian Bureau of Statistics (ABS) Cause of Death unit record files [59] (1-Jan-2006 to 30-Nov-2017) and the NSW Admitted Patient Data Collection [60] (1-Jul-2001 to 30-Jun-2018) by the NSW Centre for Health Record Linkage (CHeReL); linkage is known to be highly accurate (false-positive and false-negative rates < 0.5%). All death registrations in Australia were captured through linked data. To define study exclusions and conduct sensitivity analyses, hospitalisation records from the NSW Admitted Patient Data Collection were used. Secure data access was provided through the Sax Institute’s Secure Unified Research Environment (SURE).

Summary data on the number of deaths by selected causes of death in the Australian population in 2019, by sex and age group, were provided by the Australian Institute of Health and Welfare (AIHW) for modelling; data are from AIHW National Mortality Database. Under the methods of the Australian Burden of Disease Study, deaths that were not appropriate or valid causes of death for burden of disease analysis were redistributed by AIHW using statistical algorithms [61].

### Exposure

Smoking status was classified as “never”, “former” and “current” according to responses on the baseline questionnaire, as described previously [62]. Among those currently smoking, smoking intensity was based on the question about how much they smoked on average each day and was categorised as ≤ 14, 15–24 and ≥ 25 cigarettes/day (Additional file 1: Table S2). Age at smoking cessation among those who stopped smoking before the age of 55 was categorised as < 35, 35–44 and 45–54 years [57].

### Outcome ascertainment and definition

The outcome was cause-specific mortality. Underlying cause of death from diseases of the respiratory system, circulatory system and neoplasms, as well as some selected other conditions, was used. Common causes of death (with at least 30 deaths in total and at least 10 for each category of smoking status) as well as conditions established as caused by smoking [2–4, 7] were presented separately, with less common causes of death grouped into a category of “other”.

Participants were followed up until 30 November 2017, the latest date for linked cause of death data, or until date of death, whichever was earliest. The underlying causes of death, coded using the ICD-10 code were classified into 28 distinct cause groups as being from diseases of the respiratory system (chronic lung disease, lower respiratory infections and other diseases of the respiratory system); diseases of the circulatory system (coronary heart disease, peripheral vascular disease, cerebrovascular disease and other diseases of the circulatory system); neoplasms (cancer of lung, oro-pharynx, unknown primary site, oesophagus, liver, urinary tract, pancreas, kidney, stomach, brain, prostate (among men), large intestine, ovary (among women), breast (among women), non-Hodgkin’s lymphoma, leukaemia and other neoplasms); selected other causes (dementia including Alzheimer’s disease, cirrhosis or alcoholic liver and external causes); and general category of causes not included in the selected cause-list (Additional file 1: Table S3). Deaths with heart failure (I50) as the underlying cause of death were redistributed to other causes using the proportional allocation method [63]. As the sample size reduced with further stratification, analyses of smoking intensity and age at smoking cessation were restricted to common causes of death among those established as caused by smoking: chronic lung disease, coronary heart disease, cerebrovascular disease and lung cancer.

### Statistical methods

Participants meeting the following criteria were excluded: study withdrawal since baseline (*n* = 57, 0.02%), invalid age or date of recruitment (*n* = 551, 0.21%), data linkage errors (*n* = 248, 0.09%), age below 45 years at baseline (*n* = 7, < 0.01%), missing or invalid data on smoking status (*n* = 850, 0.32%) and missing underlying cause of death (*n* = 64, 0.02%). To minimise the potential impact of changes in smoking behaviour and higher mortality in those with baseline illness (i.e. reverse causality or the “sick quitter” effect), participants with a history of CVD (*n* = 56,820, 21%) or cancer (*n* = 30,387, 11%) at baseline were excluded. A history of CVD or cancer was based on self-report and/or hospitalisations in the 5 years prior to baseline (Additional file 1: Table S4) [64]. In addition, participants with a history of hospitalisation for chronic respiratory diseases were excluded from the analyses on diseases of the respiratory system; it was not possible to exclude individuals with respiratory illness not leading to hospitalisation, because the required information was not available from the baseline questionnaire.

Cause-specific mortality rates were directly age-and-sex standardised to the 2006 NSW population, in 5-year age groups. Hazard ratios (HRs) for cause-specific mortality by smoking status (current and former versus never) were estimated according to categories of number of cigarettes smoked among participants who reported current smoking, using Cox regression. Models with age as the underlying time variable initially included sex (except for cancers of the breast, ovary and prostate); fully adjusted models additionally included region of residence, alcohol consumption, annual household income, education attainment, private health insurance and country of birth, as well as outcome specific additional adjustments for known confounders [2–4, 65], based on self-reported health and behavioural factors at baseline (Additional file 1: Table S5) [66]. Subgroup analyses according to broad age groups (45–64, 65–74 and ≥ 75 years) and sex were reported in the additional tables for four of the most common outcomes for which smoking is an established risk factor: chronic lung disease, coronary heart disease, cerebrovascular disease and lung cancer. Analyses stratifying former smokers by age at smoking cessation were conducted restricting the data to individuals who stopped smoking before reaching 55 years of age, to ensure that all participants had the opportunity to quit at these ages and reduce the “sick quitter” effect; HRs among those ceasing smoking at ages < 35, 35–44 and 45–54 years versus those who never smoked were estimated. Missing values for covariates were included in the models as separate categories.

Confounder-adjusted smoking-attributable fractions (SAFs) for each cause of death, overall and by age group (45–74 and ≥ 75 years) and sex, were estimated using the extension of the Miettinen [67, 68] formula for multilevel exposures as $SAF={\sum }_{i=0}^{2}{P}_{{c}_{i}}\left(\frac{{RR}_{i}-1}{{RR}_{i}}\right)=1-{\sum }_{i=0}^{2}{P}_{{c}_{i}}/{RR}_{i}$ where ${RR}_{i}$ is the hazard ratio estimated from the study for exposure level $i$ (with levels $i$ = 0, 1, 2 corresponding to never, past and current smoking) and ${P}_{{c}_{i}}$ is the prevalence of exposure $i$ among cases (smoking proportions among participants who died from the outcome of interest during follow-up). SAFs were calculated for specific causes of death (Additional file 1: Table S3, causes 1 to 27) with significantly higher mortality risks for current smoking compared to never smoking. Cause-specific SAFs were applied to the corresponding age-group and sex-specific number of deaths in Australia to calculate smoking-attributable deaths in 2019. Attributable deaths for all-cause mortality were based on summation of age group and sex-specific attributable deaths.

Additional sensitivity analyses were conducted: (i) additionally adjusting for BMI; (ii) additionally adjusting for physical activity; (iii) broadening the definition of “current smoking”, categorising participants who ceased smoking within the last 3 years to current smoking; (iv) broadening and relaxing the exclusions for prior respiratory illnesses in the analyses pertaining to respiratory system diseases; (v) using an alternative exposure, pack-years of smoking; (vi) analyses excluding heavy drinkers (≥ 15 drinks/week) for causes for which alcohol is a known risk factor (cirrhosis or alcoholic liver; external causes; cancer of oro-pharynx, oesophagus, liver, large intestine or breast); and (vii) rewriting the SAF formula to include prevalence of smoking in the numerator (as shown in Additional file 1: Table S6) [67–69] and replacing the prevalence of smoking in the study with population-level prevalence. All statistical tests were two-sided using a significance level of 5%, except for the tests based on Schoenfeld residuals.

## Results

After applying study exclusions, 178,169 participants (67% of the baseline survey responders) were included in the analyses. Overall, 8% reported current smoking with a median age at smoking initiation of 17 years (interquartile range IQR; 15–20) and a median of 16 (IQR, 10–23) cigarettes smoked per day, and 34% reported former smoking, with a median age at quitting of 38 years (IQR, 29–48). Compared to those who never smoked, participants who reported current smoking were, on average, younger, more likely to be men, of lower income, of lower education level and to consume ≥ 15 alcoholic drinks/week; they were also less likely to live in major cities, hold private health insurance or receive treatment for hypertension (Table 1).

**Table 1:** Sociodemographic, lifestyle and health-related factors by smoking status at baseline

	Current Smoking			Past Smoking	Never Smoked
	< 15 cig/day	≥ 15 cig/day	Total		
Total	3% (4794)	5% (8952)	8% (14,038)	34% (59,812)	59% (104,319)
Age					
45–64 years	84% (4019)	86% (7674)	85% (11,904)	70% (41,811)	71% (74,314)
65–79 years	14% (649)	13% (1181)	13% (1886)	25% (14,692)	23% (23,564)
≥ 80 years	3% (126)	1% (97)	2% (248)	6% (3309)	6% (6441)
Men	43% (2068)	51% (4541)	48% (6756)	52% (31,227)	38% (39,357)
Region of residence					
Major cities	52% (2441)	48% (4223)	49% (6816)	51% (30,061)	55% (55,810)
Inner regional	36% (1688)	37% (3238)	36% (5016)	37% (21,681)	34% (35,116)
Outer regional/remote/very remote	12% (587)	15% (1355)	14% (1989)	12% (6895)	11% (11,338)
Highest qualification					
No school certificate	12% (590)	19% (1634)	17% (2285)	11% (6389)	9% (9407)
Certificate/diploma/trade	68% (3230)	69% (6095)	69% (9494)	66% (38,887)	62% (63,641)
Tertiary	19% (911)	12% (1072)	15% (2029)	23% (13,664)	29% (29,768)
Household income					
< $20,000	25% (984)	32% (2295)	30% (3359)	19% (9398)	19% (15,501)
$20,000–$39,999	23% (911)	23% (1657)	23% (2614)	21% (10,281)	20% (16,306)
$40,000–$69,999	25% (969)	24% (1708)	24% (2731)	25% (12,065)	24% (19,380)
$70,000 or more	26% (1019)	21% (1514)	23% (2575)	34% (16,526)	37% (30,433)
Private health insurance (hospital/DVA)	50% (2395)	39% (3502)	43% (6020)	65% (38,853)	71% (73,904)
Alcoholic drinks per week					
None	30% (1389)	34% (2966)	33% (4448)	22% (13,183)	37% (37,325)
1–14	50% (2363)	39% (3391)	43% (5876)	55% (32,355)	55% (56,030)
15 or more	20% (930)	27% (2377)	25% (3357)	23% (13,599)	8% (8563)
Physical activity tertile					
Low	30% (1379)	35% (3066)	34% (4538)	28% (16,017)	30% (30,361)
Medium	33% (1536)	30% (2598)	31% (4211)	34% (19,806)	35% (35,802)
High	37% (1701)	34% (2979)	35% (4768)	38% (22,393)	35% (34,884)
Physical functioning limitation (MOS-PF < 75)	16% (664)	23% (1783)	21% (2496)	15% (7762)	13% (11,727)
Body mass index					
15 to < 18.5 kg/m^2^	3% (121)	3% (211)	3% (342)	1% (440)	1% (1142)
18.5 to < 20 kg/m^2^	5% (210)	4% (340)	4% (564)	2% (1155)	3% (3019)
20 to < 25 kg/m^2^	41% (1810)	36% (2983)	38% (4892)	31% (17,281)	37% (35,812)
25 to < 30 kg/m^2^	35% (1539)	35% (2863)	35% (4479)	42% (23,215)	39% (37,210)
30 to 50 kg/m^2^	17% (773)	22% (1846)	21% (2674)	24% (13,613)	20% (19,309)
Doctor diagnosed diabetes	5% (248)	7% (650)	7% (921)	8% (4611)	6% (6387)
Treatment for hypertension	13% (626)	16% (1472)	15% (2144)	21% (12,648)	21% (21,408)
Treatment for high cholesterol	10% (456)	11% (984)	10% (1469)	13% (8020)	12% (12,205)
Taking aspirin	12% (560)	12% (1094)	12% (1690)	15% (9129)	13% (13,795)

One alcoholic drink was roughly equivalent to one standard drink containing 10 ml of alcohol. Physical activity tertiles were based on physical activity sessions per week, weighted for intensity

Percentages shown in the “Total” row represent proportions of the total study population (*n* = 178,169)

All other percentages represent proportions within smoking status categories. Denominators of the percentages do not include missing data. Number of study participants with missing data: region of residence = 3447; education = 2605; annual household income = 37,000; private health insurance = 4; alcohol consumption = 3433; physical activity tertile = 5389; physical functioning limitation = 21,721; body mass index = 13,022; taking aspirin = 17; smoking intensity = 292; there were no missing data for age or sex, and missing data were not applicable for variables related to diabetes, hypertension, and high cholesterol

*DVA* Department of Veterans’ Affairs, *MOS-PF* Medical Outcomes Study–Physical Functioning scale

Over 1.68 million person-years of follow-up (median 9.32 years), there were 13,608 deaths in the cohort, including 5138 (38%) from neoplasms, 3504 (26%) from diseases of the circulatory system and 994 (7%) from diseases of the respiratory system. Considering specific causes of death, the greatest number of deaths among those who never smoked were from coronary heart disease, cerebrovascular disease and dementia/Alzheimer’s disease, while for those who reported current smoking the greatest number of deaths were from cancer of lung, coronary heart disease and chronic lung disease.

**Fig. 1:**
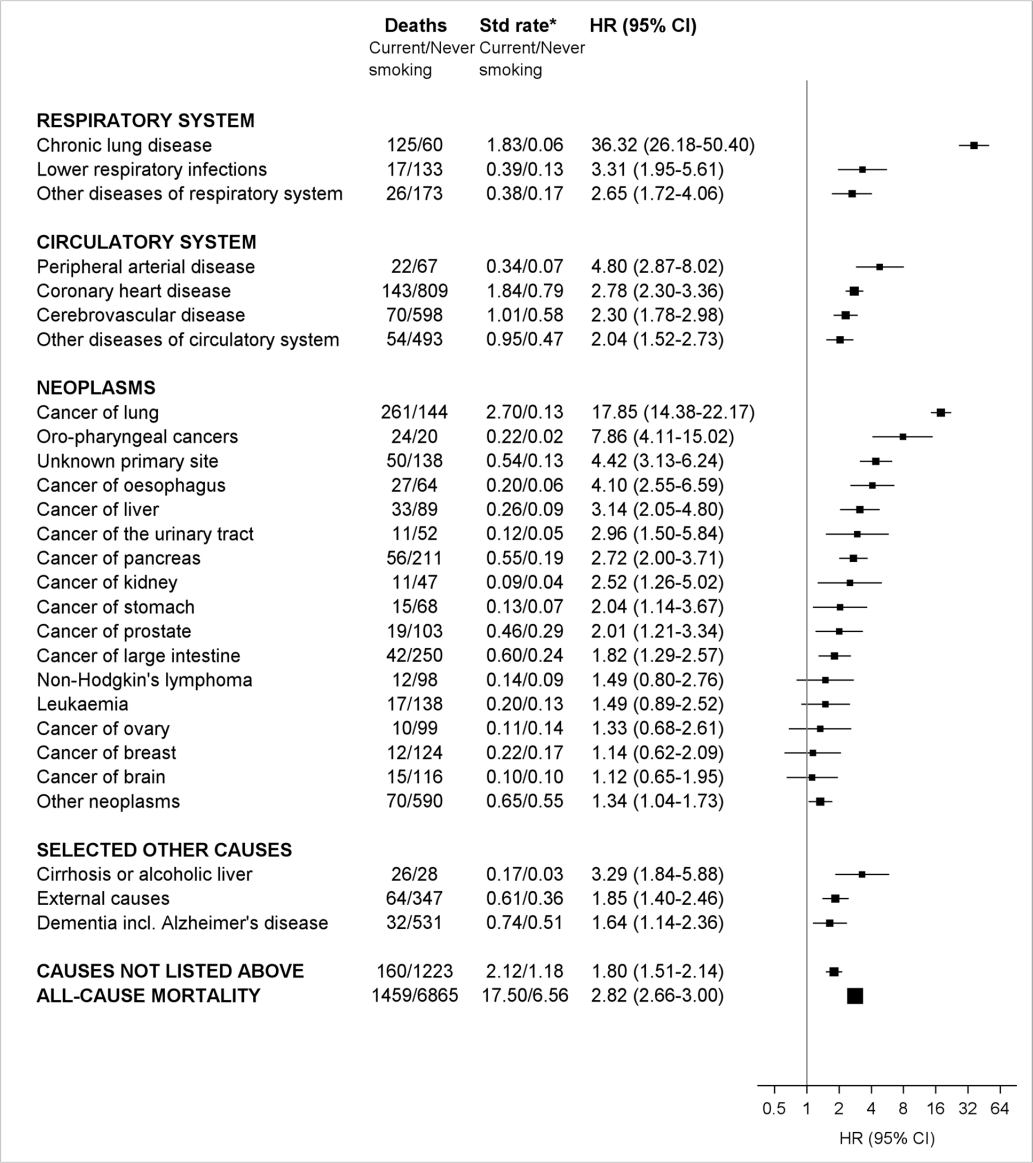
Risk of death from a range of common causes associated with current versus never smoking *Age-sex standardised rate per 1000 person-years; rates for cancer of prostate, cancer of ovary and cancer of breast are sex-specific. In addition to exclusions based on history of CVD or cancer at baseline, additional exclusions for chronic respiratory illness at baseline were applied to analyses involving diseases of the respiratory system. As a result, cause-specific numbers of deaths do not add to deaths from any cause. Hazard ratios (HR) adjusted for age as the underlying time variable, sex, region of residence, alcohol consumption, income, education, private health insurance and country of birth, with additional outcome-specific adjustments as outlined in Additional file 1: Table S5, are plotted on a log scale and are represented by squares of areas, which are inversely proportional to standard errors of current smokers’ hazard ratios. The proportionality assumption for smoking status was violated for all-cause mortality, indicating interaction by age; in analyses stratified by age group attenuation in HRs with increasing age was noted. HRs for all-cause mortality for 45–64 years, 65–74 years and ≥ 75 years were 2.75 (2.56–2.95), 2.67 (2.47–2.89) and 2.09 (1.95–2.25), respectively

Compared to never smoking, HRs associated with current smoking were significantly increased for a wide range of conditions (Fig. 1, Additional file 1: Table S7) and were very high for chronic lung disease (adjusted HR = 36.32, 95%CI = 26.18–50.40), cancer of the lung (17.85, 14.38–22.17) and cancer of mouth/pharynx/larynx/nasal-cavity/sinuses (7.86, 4.11–15.02); three to five times as high for deaths from lower respiratory infections, peripheral vascular disease, cancer of oesophagus, cancer of liver, as well as cancer of unknown primary site; and at least two-fold for other disease of the respiratory system, coronary heart disease, cerebrovascular disease and other diseases of the circulatory system, as well as for cancer of the urinary tract, pancreas, kidney, stomach and prostate. The relative risk of death from dementia (including Alzheimer’s disease) was 1.64 (1.14–2.36) with current versus never smoking.

Compared to never smoking, mortality risks generally remained elevated among those who reported former smoking at baseline to a lesser extent than current smoking, with significantly raised HRs observed for deaths from chronic lung disease, lower respiratory infections, other diseases of respiratory system, peripheral arterial disease, coronary heart disease and oro-pharyngeal cancers, as well as cancer of lung, oesophagus, urinary tract and unknown primary site.

Current smoking was associated with significantly elevated risk of death from any cause (HR current versus never smoking = 2.82, 2.66–3.00 overall); this relative risk did not vary significantly according to sex (2.75, 2.54–2.98 for men; 2.83, 2.57–3.10 for women), and point estimates for deaths from any cause were at least twofold in all age and sex groups (Additional file 1: Table S8). 

In analyses stratified by sex, HRs for current smoking were significantly higher in men for deaths from external causes and in women for deaths from dementia/Alzheimer’s disease (Additional file 1: Table S9). HRs associated with current smoking compared to never-smoking for deaths from cancer of oesophagus, stomach and liver remained significantly higher in men as in the main analysis; corresponding HRs could not be estimated for women due to the small number of deaths.

**Fig. 2:**
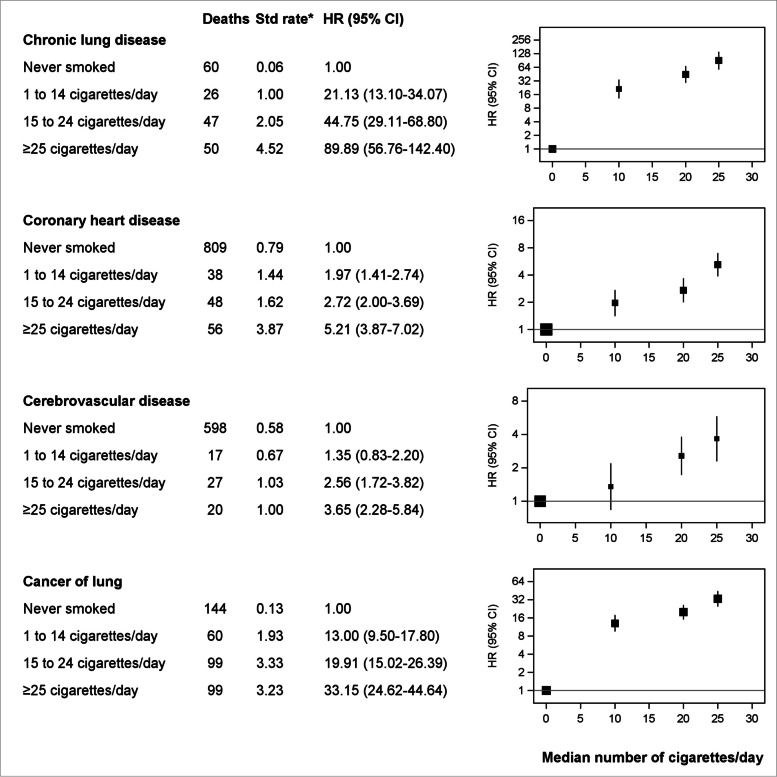
Risk of death comparing current with never smoking, according to amount smoked. Analyses were restricted to common causes of death among those established as caused by smoking: chronic lung disease, coronary heart disease, cerebrovascular disease and lung cancer. *Age-sex standardised rate per 1000 person-years. Hazard ratios (HR) are adjusted for age, sex, region of residence, alcohol consumption, annual household income, education attainment, country of birth (Australia vs. Other) and private health insurance; hazard ratios for cancer of lung were also adjusted for fruit intake. Hazard ratios represented by squares are plotted on a log scale, against the median number of cigarettes within each category reported at resurvey among those who reported being current smokers at resurvey (Additional file 1: Table S10), as this was considered the best estimate of long-term mean consumption among all in that category. Rates in never smokers were plotted against the “0” on the x-axis; areas of squares are proportional to the natural logarithm of the number of deaths

**Fig. 3:**
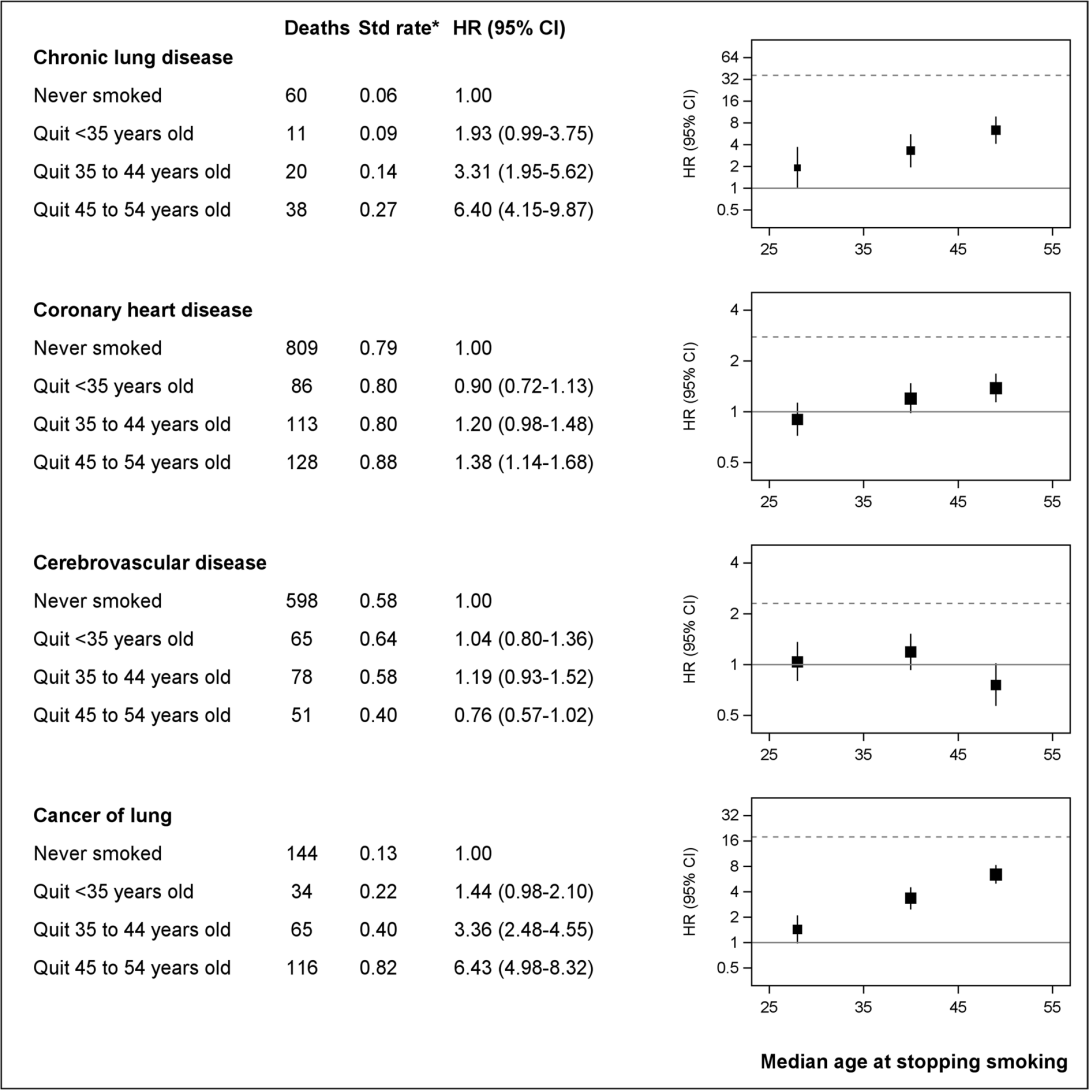
Risk of death comparing past with never smoking, according to age at smoking cessation. Analyses were restricted to common causes of death among those established as caused by smoking: chronic lung disease, coronary heart disease, cerebrovascular disease and lung cancer. *Age-sex standardised rate per 1000 person-years. Hazard ratios (HR) as in Fig. 1

For the four most common causes of death (chronic lung disease, cancer of the lung, coronary heart disease and cerebrovascular disease), mortality risk increased with increasing smoking intensity and excess risks were largely avoided with cessation before age 45 years (Figs. 2, 3). In Fig. 2, hazard ratios are plotted on a log scale against the median number of cigarettes within each category reported at resurvey among those who reported being current smokers at resurvey (Additional file 1: Table S10).

Estimates did not change materially in sensitivity analyses with additional adjustment for BMI (Additional file 1: Table S11) or physical activity (Additional file 1: Table S12), reclassifying those who quit smoking in the 3 years prior to baseline as current smokers (Additional file 1: Table S13), varying exclusions for respiratory diseases at baseline (Additional file 1: Table S14), or using pack-years of smoking as exposure (Additional file 1: Table S15). Restricting analyses to non-drinkers or people consuming < 15 drinks/week weakened smoking associated risk for external causes and the cancers considered; however, all except that for breast cancer remained significantly elevated (Additional file 1: Table S16).

**Fig. 4:**
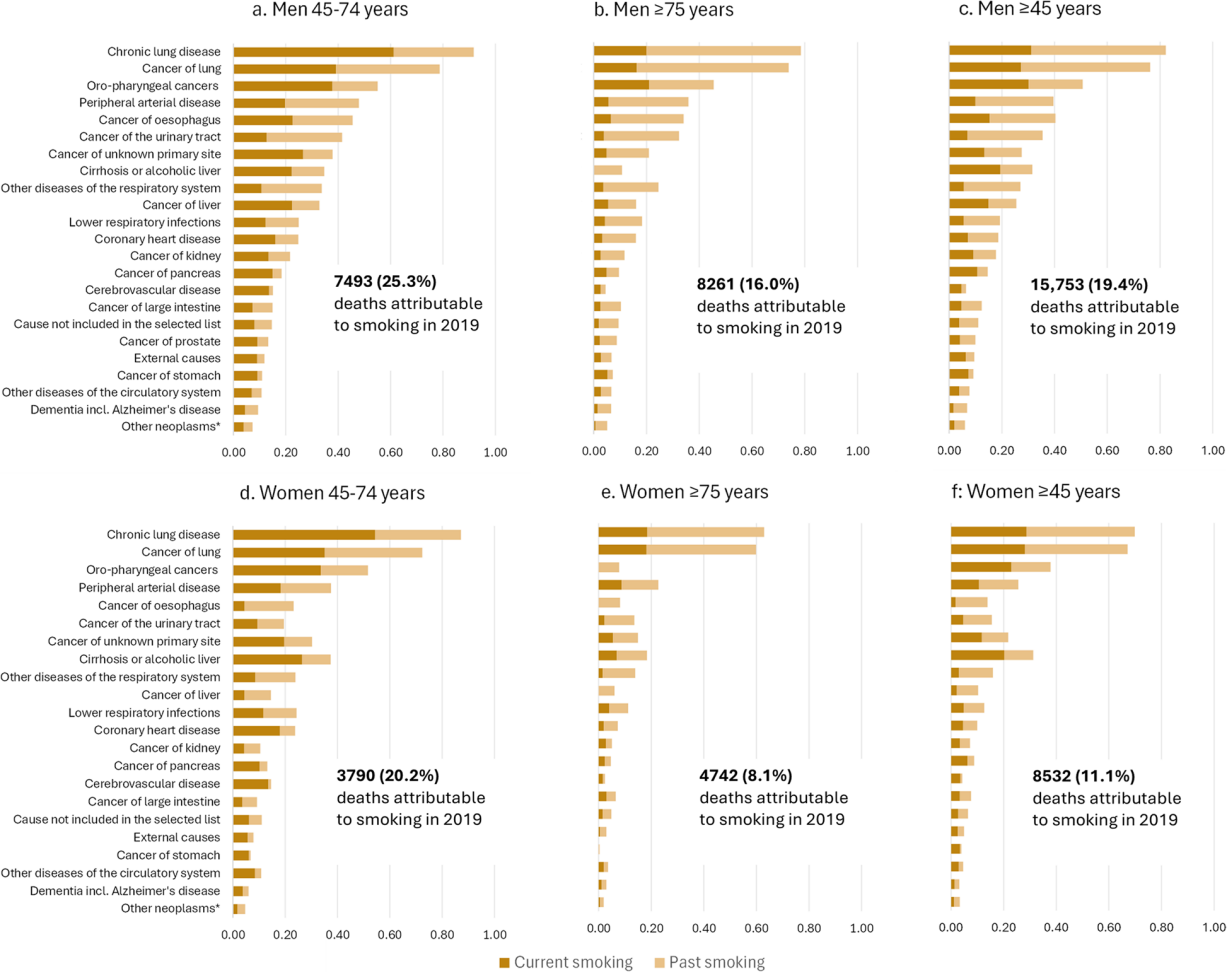
Smoking-attributable fractions for current and past smoking by age group and sex in Australia. A total of 24,285 (15.3%) deaths in the Australian population in 2019—11,283 (23.3%) among 45–74-year-olds and 13,002 (11.8%) among ≥ 75-year-olds—were attributable to smoking. As current smoking was not associated in these analyses with significantly increased risk of death from non-Hodgkin’s lymphoma, leukaemia or cancers of ovary, breast and brain, they were not included in the calculation

A total of 24,285 (15.3%) deaths at age ≥ 45 years in 2019—11,283 (23.3%) among 45–74-year-olds and 13,002 (11.8%) among ≥ 75-year-olds—were attributable to current or past smoking (Fig. 4, Additional file 1: Table S17). Attributable deaths were nearly double in men than among women: 15,753 (19.4%) versus 8532 (11.1%). Smoking attributable deaths at age 45–74 were largely due to current smoking (59.1%) while smoking attributable deaths at age 75 or above were largely due to past smoking (72.8%) (Fig. 4, Additional file 1: Table S17). Overall, smoking-attributable fractions varied by age group and sex from around 63% to 92% for deaths from chronic lung disease and around 3% to 9.5% for deaths from dementia (Fig. 4, Additional file 1: Table S18). Attributable fractions were generally higher for those aged 45–74 years versus ≥ 75 years and for men versus women. Neoplasms featured prominently in smoking-attributable fractions, accounting for 7311 smoking-attributable deaths in men and 3334 deaths in women, closely followed by diseases of the respiratory system (4175 deaths in men and 3112 deaths in women) and diseases of the circulatory system (3125 deaths in men and 1482 deaths in women) (Fig. 4, Additional file 1: Tables S17, S18). Over three-quarters (79%) of deaths in men from lung cancer and chronic lung disease were attributable to smoking (3900 and 3271 attributable deaths, respectively); the corresponding proportion was 69% in women (2377 and 2517 attributable deaths, respectively). Although smoking-attributable fractions were relatively smaller for coronary heart disease (18.6% for men and 9.9% for women), it was the third leading cause of attributable deaths (2003 deaths in men and 708 deaths in women). Around one-third of deaths among those aged 45–74 years with cirrhosis or alcoholic liver disease as the underlying cause of death were attributable to current or past smoking. Smoking-attributable fractions estimated using population-level prevalence of smoking were higher compared to those using smoking prevalence from the study (17.5% versus 15.3% overall), especially for 45–74-year-old-men (30% versus 25.3%, Additional file 1: Table S19).

## Discussion

Smoking has a continuing substantial impact on mortality in Australia, despite world-leading tobacco control. In 2019, around 1-in-5 (19.4%) deaths among men and around 1-in-9 (11.1%) deaths among women aged 45 years and over were attributable to current or past smoking, amounting to around 24,000 deaths per year. Moreover, nearly one-quarter of premature deaths—23.3% of deaths at age 45–75 years—can be attributed to current or past smoking.

This large population-based study provides the most comprehensive and contemporary evidence on the impact of smoking on cause-specific mortality in Australia (based on formal searches outlined in Additional file 2: Rapid review of evidence on smoking and cause-specific mortality), which is of relevance to many other countries with a mature tobacco epidemic. Current smokers had elevated mortality rates from virtually all causes, including at least a two-fold increase in risk of death from respiratory and circulatory disease, a 60% increased risk of death from dementia (including Alzheimer’s disease) and considerable increases in risk of death from cancer. The greatest increase in relative risk was observed for deaths from chronic lung disease, lung cancer and cancers of the oro-pharynx, with death rates in people who smoke of 36, 18 and 8 times those of never smokers, respectively. The majority of deaths from these causes were attributable to current smoking. Risks of each cause of death were substantially elevated for lower-intensity smoking (i.e. 1–14 cigarettes per day) and increased with increasing smoking intensity. Former smokers generally had lower mortality rates compared to those who continued to smoke, and smoking-related risks associated with each cause of death were largely avoided if cessation occurred before the age of 45 years.

The large sample allowed investigation into the risks of smoking for 28 specific causes of death, which had at least 30 deaths in the cohort. Risks for causes with fewer cases (e.g. pulmonary fibrosis, bladder cancer and multiple myeloma), which included some causes for which smoking might be protective (e.g. endometrial cancer, ulcerative colitis and Parkinson’s disease), could not be quantified as the available data were insufficient for meaningful comparisons. While the 45 and Up Study is not designed to be representative of the general population, relative effect estimates based on internal comparisons are likely to be valid. Attributable fractions were estimated using adjusted hazard ratios, in keeping with best practice. Detailed questionnaire data on smoking behaviour allowed quantification of the risks associated with smoking intensity and evidence on the benefits of smoking cessation in relation to age at quitting. We were able to minimise the impact of smoking cessation due to ill-health, or “reverse causality”, by using questionnaire and linked administrative data to exclude people with prior CVD and cancer.

The available data allowed adjustment for a wide range of factors associated with both smoking and mortality outcomes, including age, sex, socioeconomic status and a range of self-reported health and behavioural factors such as alcohol consumption, cancer screening and dietary factors. Smoking-related risks did not change substantively following adjustment for all outcomes, except for death from cirrhosis and alcoholic liver disease—largely due to adjustment for alcohol consumption. Mortality from external causes was also raised in people who smoke. Given the systemic effects of smoking and its potential contribution to falls, mental health problems and risk-taking behaviour, some element of causality remains plausible, but the extent of this is unclear. Hence for certain less common outcomes, such as mortality from cirrhosis, alcoholic liver disease and external causes, the relationship with smoking may be partly or largely non-causal. Considered in perspective, the current evidence indicates that the bulk of the excess mortality observed in people who smoke is likely to be caused by smoking [2–4].

Deaths from external causes were included in the calculation of SAFs out of public health precaution, since underestimation of risk has adverse implications, noting that smoking-attributable deaths from external causes (794, 0.5%) had minimal impact on the overall estimate. The underlying cause of death alone may be less reliable in old age, because of multimorbidity at death and potential misclassification. Our study includes community-dwelling individuals aged 45 or over, and deaths at younger ages are not included in attributable fractions. However, estimates from the Australian Burden of Disease study indicate that relatively few deaths are attributable to tobacco use in younger age groups (zero for under 35 age group and approximately 160 deaths for 35–44 age group in 2018) [13]. By using relative risk data from an Australian population-based cohort study and by accounting for many sociodemographic factors and health-related behaviours, we are likely to be providing more accurate estimates of risk for Australia than previously available.

The findings here contribute to the worldwide detailed evidence on smoking and cause of death, with hazard ratio estimates largely consistent with previous studies on cancer, cardiovascular disease and respiratory disease outcomes [6, 18, 26, 33, 49], especially for deaths from chronic lung disease and other established smoking-related causes of death [3]. Also we generally observed null results for causes of death that are not considered to be smoking-related cancers [7] such as non-Hodgkin’s lymphoma and leukaemia. Most studies reporting cancer-specific mortality estimates focus on all cancers combined, lung cancer or smoking-related cancers combined, supporting the value of the data presented here [7]. Our results are consistent with evidence from previous large-scale studies which indicate increased risks of death from external causes, alcoholic poisoning and alcoholic liver disease among people who smoke [30, 33].

Dementia is a leading cause of death in many countries and our study indicates a moderately greater risk of dying from dementia with current compared to never smoking. While smoking is a generally accepted risk factor for developing vascular and Alzheimer’s dementia [70], evidence is limited and this is the first study, to our knowledge, showing an increased risk of dying from dementia (including Alzheimer’s disease) with current- versus never smoking, based on large-scale evidence. This finding should be interpreted with caution, particularly as it has not been consistently observed, noting null findings for smoking and death from dementia in the UK Million Women Study. Moreover, it was not possible to separate death from vascular dementia, Alzheimer’s and other types of dementia in the data provided for this analysis, and risk factors can differ according to subtype [4].

Australia has a mature smoking epidemic, heavy and prolonged smoking among older current smokers, and low death rates in never smokers, leading to relative risk mortality estimates for current versus never smoking which are generally higher than average international estimates. Since relative risks are known to vary between countries and over time, direct, local relative risk estimates are more accurate and were used in our study, leading to an estimate of 24,285 (15.3%) smoking-attributable deaths in 2019. This is higher than previous estimates based on average international relative risks, including AIHW estimates of 20,500 deaths (13%) in 2018 [12, 13] and a Global Burden of Disease Study estimate of around 12,000 deaths (7.25%) among those aged 25 years and over in 2019 [14].

Although a dose–response of increasing mortality with increasing smoking intensity is well-established, large-scale evidence on the risks of low-intensity smoking is very limited. Furthermore, many people who smoke underestimate the impact of smoking relatively few cigarettes per day [71], and less frequent smoking, particularly with the rise in combined smoking and novel nicotine product use (e.g. electronic cigarettes, nicotine pouches), remains an important public health challenge. Our findings of 13- and 20-fold risks of dying of lung cancer and chronic lung disease, respectively, and a doubling in coronary heart disease mortality with 1–14 cigarettes/day (median 10 cigarettes/day) is important policy-relevant information and adds substantively to the global emerging consistency of available evidence [4, 5, 24, 39, 46].

## Conclusions

Tobacco remains Australia’s leading cause of premature death. However, the tobacco industry seeks to combat tobacco control, including through misinformation about tobacco products and political lobbying to influence public health policymaking [72]. This, along with other factors, means that investment in tobacco control—and perceptions of harm [73], including among smokers—are not commensurate with the impact of smoking [74]. Timely and accurate mortality estimates demonstrating large ongoing impacts on mortality should contribute to acceleration in tobacco control, through extended and more effective implementation of effective measures, including those outlined in the WHO Framework Convention on Tobacco Control. This includes greater whole-of-population and priority population initiatives.

## Supplementary Information

Additional file 1: Table S1. Previous estimates of smoking attributable deaths in Australia. Table S2. Exposure definitions. Table S3. Outcome definitions. Table S4. Definitions used for history of diseases. Table S5. Adjustments for potential confounding and modelling strategy. Table S6. Calculation of smoking-attributable fractions. Table S7. Risk of death from common causes for current and past versus never smoking. Table S8. Risk of death from selected causes by age and sex for current and past versus never smoking. Table S9. Sex specific HRs, where interaction between sex and smoking status was identified in main analysis. Table S10. Smoking patterns at resurvey by smoking status at recruitment. Table S11. Hazard ratios for current versus never smoking: Sensitivity analysis, with additional adjustment for BMI. Table S12. Hazard ratios for current and past versus never smoking: Sensitivity analysis, with additional adjustment for physical activity tertile. Table S13. Deaths, death rates and hazard ratios for current and past versus never smoking: Sensitivity analysis broadening the definition of “current smoking”. Table S14. Deaths, death rates and hazard ratios for deaths from diseases of the respiratory system, with varying exclusions for respiratory diseases at baseline. Table S15. Deaths, death rates and hazard ratios for current versus never smoking: Sensitivity analysis using pack-years of smoking as the exposure. Table S16. Hazard ratios for current versus never smoking: Sensitivity analysis excluding heavy drinkers (≥ 15 drinks/week), for causes for which alcohol is an important risk factor. Table S17. Estimates of smoking attributable deaths in Australia, by age group and sex, 2019. Table S18. Estimates of smoking-attributable fractions of death for current and past smoking, by age and sex. Table S19. Estimates of smoking-attributable fractions using within-study versus population prevalence of smoking.

Additional file 2. Literature review.

## Data Availability

This research was completed using data collected through the 45 and Up Study (www.saxinstitute.org.au). The study questionnaire is available at https://www.saxinstitute.org.au/our-work/45-up-study/questionnaires/. Data supporting the findings from this study are available from the Sax Institute, the NSW Department of Health and the Australian Bureau of Statistics, with data linkage conducted by the NSW Centre for Health Record Linkage. Restrictions apply to the availability of these data, which were used under licence for the current study, and so are not publicly available. Data are however available from the authors upon reasonable request and with permission of the Sax Institute (www.saxinstitute.org.au) and the NSW Department of Health. Summary data on the number of cause-specific deaths in Australia (used in the estimation of population attributable deaths) were provided by the AIHW. Cause of Death Unit Record File data are provided to the AIHW by the Registries of Births, Deaths and Marriages and the National Coronial Information System (managed by the Victorian Department of Justice) and include cause of death coded by the Australian Bureau of Statistics (ABS). The data are maintained by the AIHW in the National Mortality Database.

## Abbreviations

ABS: Australian Bureau of Statistics
CHeReL: Centre for Health Record Linkage
SURE: Secure Unified Research Environment
AIHW: Australian Institute of Health and Welfare
HRs: Hazard ratios
SAFs: Smoking-attributable fractions
IQR: Interquartile range
CVD: Cardiovascular disease
NSW: New South Wales
DVA: Department of Veterans’ Affairs
MOS-PF: Medical Outcomes Study–Physical Functioning scale
