# Supplementary material for: Relationship of tobacco smoking to cause-specific mortality: contemporary estimates from Australia
Source: BMC Med. 2025 Feb 25;23:115. doi: 10.1186/s12916-025-03883-9 (PMC11854379; doi:10.1186/s12916-025-03883-9)
Supplement: Supplementary file 1 — Additional file 1: Table S1. Previous estimates of smoking attributable deaths in Australia. Table S2. Exposure definitions. Table S3. Outcome definitions. Table S4. Definitions used for history of diseases. Table S5. Adjustments for potential confounding and modelling strategy. Table S6. Calculation of smoking-attributable fractions. Table S7. Risk of death from common causes for current and past versus never smoking. Table S8. Risk of death from selected causes by age and sex for current and past versus never smoking. Table S9. Sex specific HRs, where interaction between sex and smoking status was identified in main analysis. Table S10. Smoking patterns at resurvey by smoking status at recruitment. Table S11. Hazard ratios for current versus never smoking: Sensitivity analysis, with additional adjustment for BMI. Table S12. Hazard ratios for current and past versus never smoking: Sensitivity analysis, with additional adjustment for physical activity tertile. Table S13. Deaths, death rates and hazard ratios for current and past versus never smoking: Sensitivity analysis broadening the definition of “current smoking”. Table S14. Deaths, death rates and hazard ratios for deaths from diseases of the respiratory system, with varying exclusions for respiratory diseases at baseline. Table S15. Deaths, death rates and hazard ratios for current versus never smoking: Sensitivity analysis using pack-years of smoking as the exposure. Table S16. Hazard ratios for current versus never smoking: Sensitivity analysis excluding heavy drinkers (≥ 15 drinks/week), for causes for which alcohol is an important risk factor. Table S17. Estimates of smoking attributable deaths in Australia, by age group and sex, 2019. Table S18. Estimates of smoking-attributable fractions of death for current and past smoking, by age and sex. Table S19. Estimates of smoking-attributable fractions using within-study versus population prevalence of smoking. [file 12916_2025_3883_MOESM1_ESM.pdf]

Relationship of tobacco smoking to cause-specific mortality: Contemporary estimates from Australia

Additional File 1. Tables S1-S19

Contents

Table S1. Previous estimates of smoking attributable deaths in Australia 2
Table S2. Exposure definitions 3
Table S3. Outcome definitions........ 4
Table S4. Definitions used for history of diseases........ 5
Table S5. Adjustments for potential confounding and modelling strategy........ 6
Table S6. Calculation of smoking-attributable fractions 8
Table S7. Risk of death from common causes for current and past versus never smoking........ 9
Table S8. Risk of death from selected causes by age and sex for current and past versus never smoking 10
Table S9. Sex specific HRs, where interaction between sex and smoking status was identified in main analysis 12
Table S10. Smoking patterns at resurvey by smoking status at recruitment 13
Table S11. Hazard ratios for current versus never smoking: Sensitivity analysis, with additional adjustment for BMI 14
Table S12. Hazard ratios for current and past versus never smoking: Sensitivity analysis, with additional adjustment for physical activity tertile........ 15
Table S13. Deaths, death rates and hazard ratios for current and past versus never smoking: Sensitivity analysis broadening the definition of "current smoking" to include those who quit 3 years prior to baseline 16
Table S14. Deaths, death rates and hazard ratios for deaths from diseases of the respiratory system, with varying exclusions for respiratory diseases at baseline 17
Table S15. Deaths, death rates and hazard ratios for current versus never smoking: Sensitivity analysis using pack-years of smoking as the exposure 18
Table S16. Hazard ratios for current versus never smoking: Sensitivity analysis excluding heavy drinkers (≥15 drinks/week), for causes for which alcohol is a known risk factor 19
Table S17. Estimates of smoking attributable deaths in Australia, by age group and sex, 2019 20
Table S18. Estimates of smoking-attributable fractions of death for current and past smoking, by age and sex 21
Table S19. Estimates of smoking-attributable fractions using within-study versus population prevalence of smoking 22

Table S1. Previous estimates of smoking attributable deaths in Australia

Applying estimates of aetiological fractions published in 1995 [8] and revised in 2001 [9] 14,901 (11%) deaths in Australia in 2004-05 were attributed to tobacco smoking [10].
Applying the Peto method, using World Health Organization (WHO) mortality data for lung cancer and for other diseases, an estimated 16% (23,000) deaths in Australia were attributable to smoking in 2010 [11].
According to more recent estimates from the Australian Burden of Disease Study (ABDS) using a comparative risk assessment method, tobacco use contributed to 20,933 (13%) deaths in 2015 and 20,482 (12.9%) in 2018 [12,13]. The ABDS used a smoking impact ratio method to quantify the contribution of tobacco use to various types of cancers and chronic respiratory conditions, whereas current/former smoking prevalence from the National Drug Strategy Household Survey (5-year lagged) and relative risks from the Global Burden of Disease Study (combined with Australian Institute of Health and Welfare literature search) were used to estimate population attributable fractions (PAFs) for other conditions.
Using a comparative risk assessment framework, the Global Burden of Disease Study 2021 estimated deaths due to smoking in Australia to be around 12,000 (7%) among those aged 25 years and over in 2019 [14].

Reference numbers refer to the reference list in the main manuscript.

Table S2. Exposure definitions

#	Domain	Question	Response options	Categorisation
1	Smoking status	Have you ever been a regular smoker?	Yes<br>No	Never smokers: "No"
2		If "Yes" to Q1, how old were you when you started smoking regularly?	Years old	NA
3		Are you a regular smoker now?	Yes<br>No	Current smokers: "Yes" to Q1 and Q3<br>Past smokers: "Yes" to Q1, "No" to Q3
4	Age cessation of smoking	If "No", how old were you when you stopped smoking regularly?	Years old	Categorised as:<br>• <25 years<br>• 25-34 years<br>• 35-44 years<br>• 45-54 years<br>• ≥55 years
5	Smoking intensity	About how much do you/ did you smoke on average each day?	Cigarettes<br>Pipes and cigars	Categorised as:<br>• ≤14 cigarettes/day<br>• 15-24 cigarettes/day<br>• ≥25 cigarettes/day

Smoking status was determined based on responses to Q1, Q2, and Q3. The age of cessation (Q4) and smoking intensity (Q5) were further categorised as indicated for more detailed analysis among those who reported current and past smoking. Smoking intensity categories were chosen to allow approximately one-third of participants in each category. Further information regarding the study questionnaires is available at: https://www.saxinstitute.org.au/solutions/45-and-up-study/use-the-45-and-up-study/data-and-technical-information/

Table S3. Outcome definitions

Causes of Death		ICD 10 codes
RESPIRATORY SYSTEM		
1	Chronic lung disease	J40–J44
2	Lower respiratory infections	J09-J18, J20-J22, J85-J86
3	Other diseases of respiratory system	J00-J99 except chronic lung disease or lower respiratory infections
CIRCULATORY SYSTEM		
4	Peripheral arterial disease (PVD)	I70-I74
5	Coronary heart disease (CHD)	I20-I25
6	Cerebrovascular disease (CereV)	I60–I69
7	Other diseases of circulatory system	I00-I99 except PVD, CHD, CereV
NEOPLASMS		
8	Cancer of lung	C33-C34
9	Oro-pharyngeal cancers (cancer of mouth, pharynx, larynx, nasal cavity, or sinuses)	C00–C14, C30–C32
10	Cancer of unknown primary site	C80
11	Cancer of oesophagus	C15
12	Cancer of liver	C22
13	Cancer of urinary tract	C66-C68
14	Cancer of pancreas	C25
15	Cancer of kidney	C64
16	Cancer of stomach	C16
17	Cancer of prostate	C61
18	Cancer of large intestine	C18-20
19	Non-Hodgkin's lymphoma	C82-C86
20	Leukaemia	C91-C95
21	Cancer of ovary	C56
22	Cancer of breast	C50
23	Cancer of brain	C71
24	Other neoplasms	C00-C99, D00-D89 other than neoplasms defined above
SELECTED OTHER CAUSES		
25	Cirrhosis or alcoholic liver	K70-K74
26	External causes	V00-Y98
27	Dementia including Alzheimer's disease	F01, F03, G30
ALL OTHER CAUSES		
28	Causes not included in the selected list	Causes other than respiratory diseases, diseases of the circulatory system, neoplasms and selected other causes

The following causes of death that were initially considered but combined with the relevant "other" category due to small number of deaths: gallbladder and extrahepatic bile ducts (C23-C24), mesothelioma (C45), uterine cancer (C54-C55), myelodysplasia (D46), diabetes (E10–14), motor neurone disease (G12.2), intestinal ischaemia (K55), ulcerative colitis (K51) and Parkinson's disease (G20).

Table S4. Definitions used for history of diseases

	**Definition**
History of cancer	Self-report (doctor-diagnosed cancer other than non-melanoma skin cancer) on the baseline questionnaire or hospital admission for cancer in the 5 years prior to baseline [primary or additional ICD-10 diagnoses codes C00–C97 (except C44 codes), D45, D46, D47.1, D47.3–D47.5; principal diagnosis is a cancer-related treatment (ICD-10-AM codes Z08, Z40.00, Z40.01, Z51.0, Z51.1, Z54.1, Z54.2)].
History of CVD	Self-reported doctor-diagnosed heart disease, stroke or blood clot on the baseline questionnaire or hospital admission for major CVD in the 5 years prior to baseline. Major CVD was previously defined in Joshy et.al. (66).
History of chronic respiratory diseases	Hospitalisation for chronic respiratory diseases in the 5 years prior to baseline (ICD-10-AM codes J40-J44, J47 based on all 51 diagnosis code fields in the hospital data.

Table S5. Adjustments for potential confounding and modelling strategy

Outcome	Adjustments
All outcomes	Age as the underlying time variable, sex (except for cancers of the breast, ovary and prostate), region of residence (major cities, inner regional areas, remote areas), alcohol consumption (0, 1-14, ≥15 drinks/week), annual pre-tax household income (AUD <$20,000, $20,000-$39,999, $40,000-$69,999, ≥$70,000), education attainment (No school certificate, Certificate/diploma/trade, University degree), private health insurance (yes/no) and country of birth (Australia/Other).
Cancer of lung and oro-pharyngeal cancers	*Additionally adjusted for fruit intake and vegetable intake.
Cancer of oesophagus	Additionally adjusted for aspirin use (yes/no).
Cancer of stomach	*Additionally adjusted for processed meat intake.
Cancer of liver	Additionally adjusted for use of oral or other hormonal contraceptives among women (ever use - yes/no).
Cancer of large intestine	*Additionally adjusted for fruit intake, vegetable intake, red meat intake, processed meat intake, bowel screening (ever screen – yes/no) and family history of bowel cancer (yes/no).
Cancer of prostate	Additionally adjusted for prostate specific antigen testing (ever testing – yes/no) and family history of prostate cancer (yes/no).
Cancer of breast	Additionally adjusted for parity, age at birth of first child (<25 years, ≥25 years), total duration of breastfeeding (<10 months, ≥10 months), been through menopause (yes/no), use of oral or other hormonal contraceptives (ever use - yes/no), use of hormonal therapy for the menopause (ever use - yes/no), breast screening mammography (ever screen – yes/no) and family history of breast cancer (yes/no).
Cancer of ovary	Additionally adjusted for parity, age at birth of first child (<25 years, ≥25 years), total duration of breastfeeding (<10 months, ≥10 months), been through menopause (yes/no), use of oral or other hormonal contraceptives (ever use - yes/no), use of hormonal therapy for the menopause(ever use - yes/no) and family history of ovarian cancer (yes/no).

**Dietary habits were determined based on responses to questions about usual frequency of consumption, as outlined below. These questions have been validated to estimate intake (68).*

- ***Meat intake:*** *Assessed using the question 'About how many times each week do you eat…', categories were 1) beef, lamb or pork; 2) chicken, turkey or duck; 3) processed meat including bacon, sausages, salami, devon, burgers; and 4) fish or seafood. Responses to Category 1 were used to create a flag variable for red meat intake of 5 or more times per week. Responses to category 3 were used to create a flag variable for processed meat intake of 1 or more times per week.*
- ***Vegetable intake:*** *Assessed using the question 'About how many serves of vegetables do you usually eat each day? A serve is half a cup of cooked vegetables or one cup of salad (please include potatoes and put "0" if less than one a day)…', and response options were 1) number of serves of cooked vegetables each day; 2) number of serves of raw vegetables each day (e.g. salad); and 3) I don't eat vegetables (tick box). Responses to categories 1 and 2 were combined to create a flag variable for intake of 5 or more serves of vegetables per day.*
- ***Fruit intake:*** *Assessed using the question 'About how many serves of fruit or glasses of fruit juice do you usually have each day? A serve is 1 medium piece or 2 small pieces or 1 cup of diced or canned fruit pieces*

(put "0" if you eat less than one serve a day)...', and response options were 1) number of serves of fruit each day; 2) number of glasses of fruit juice each day; and 3) I don't eat fruit (tick box). Responses to category 1 was used to create a flag variable for intake of at least 2 serves of fruit per day.

Further information regarding the study questionnaires is available at:
https://www.saxinstitute.org.au/solutions/45-and-up-study/use-the-45-and-up-study/data-and-technical-information/

Modelling strategy: *Hazard ratios (HRs) for cause-specific mortality by smoking status (current and former vs. never) were estimated according to categories of number of cigarettes smoked among participants who reported current smoking, using Cox regression. Models with age as the underlying time variable, initially included sex (except for cancers of the breast, ovary and prostate); fully adjusted models additionally included region of residence, alcohol consumption, annual household income, education attainment, private health insurance and country of birth, as well as outcome specific additional adjustments. Men and women were initially modelled together. For models with significant interaction between the main exposure and sex, results by sex are reported in Table S9. The proportionality assumption for the Cox regression was verified by tests based on weighted Shoenfeld residuals with a significance level set at 0.00001 for each model due to the large sample size; a stratified form was used where covariates displayed non-proportionality of hazards.*

Table S6. Calculation of smoking-attributable fractions

Main analysis: Confounder-adjusted smoking-attributable fractions (SAFs) for each cause of death, overall and by age group (45-74 and ≥75 years) and sex, were estimated using the extension of the Miettinen [Miettinen 1974, Khosravi 2021] formula for multilevel exposures as $SAF = \sum_i P_{c_i}\left(\frac{RR_i-1}{RR_i}\right) = 1 - \sum_i P_{c_i}/RR_i$ where RR_i is the adjusted hazard ratio estimated from the study for exposure level i (with smoking levels i = 0, 1, 2 corresponding to never, past and current smoking) and P_{c_i} is the prevalence of exposure i among cases (smoking proportions among participants who died from the outcome of interest during follow-up).

SAFs were calculated for specific causes of death (Table S3, causes 1 to 27) with significantly higher mortality risks for current smoking compared to never smoking. Specifically for our study, $SAF = \sum_{i=0}^{2} P_{c_i}\left(\frac{RR_i-1}{RR_i}\right) = 1 - \sum_{i=0}^{2} P_{c_i}/RR_i$ where RR_i is the confounder adjusted hazard ratio estimated from the study for exposure level i (current and past smoking) compared with never smoking, and P_{c_i} is the prevalence of exposure i among cases (people who died during follow-up).

Sensitivity analysis: The formulae used in the main analysis to calculate SAF can be rewritten as shown below to include the study-based prevalence of smoking among cases and controls combined, $Prev_i$.

P_{c_i} is the prevalence of exposure i among cases (people who died during follow-up).

$P_{c_i} = d_i/D$ ----- Equation-1

where d_i is the number of deaths in exposure i

D is the total number of deaths in the study.

$d_i = m_i \times n_i$ where n_i is the number of participants in exposure i at baseline

m_i is the proportion of deaths in exposure category i.

$d_i = m_i \times N \times \frac{n_i}{N}$ where *N* is the total number study participants.

$d_i = m_i \times N \times Prev_i$ ----- Equation-2

where $Prev_i$ is the baseline prevalence of exposure *i.*

Sensitivity analysis included replacing the study-based prevalence of smoking in equation 2 with population based prevalence of smoking to calculate the number of expected deaths ($\bar{d_i}$), total number of expected deaths $\bar{D}$ and expected prevalence of exposure among cases $\bar{P}_{c_i}$, for use as input when estimating smoking attributable fraction, as illustrated below for deaths from coronary heart disease in 45–74-year-old males. Population level prevalence of current, past and never smoking from the Australian National Health Survey 2014-15 [ABS 2016] were used as input for population estimates (17.4%, 45.6% and 36.9% respectively for men and 13.1%, 32.4% and 54.6% respectively for women).

**Deaths from coronary heart disease in 45–74-year-old males**									
	**Estimates from the study**						**Estimates for the population**		
	d_i	n_i	$m_i=d_i/n_i$	$prev_i=n_i/N$	RR_i	P_{ci}/RR_i	*pop prev$_i$*	$\bar{d_i}$	$\bar{P}_{ci}/RR_i$
Current smoking	66	5728	0.0115	0.1026	2.78	0.0899	0.174	112	0.1332
Past smoking	98	20760	0.0047	0.3718	1.31	0.2834	0.456	120	0.3035
Never smoking	100	29347	0.0034	0.5256	1	0.3788	0.369	70	0.2322
	D=264	N=55835				0.7521		$\bar{D}$ = 302	0.6689

Study based SAF=1-0.7521=0.2479 whereas population based SAF = 1-0.6689=0.331.

Table S7. Risk of death from common causes for current and past versus never smoking

Cause of death	Events Current/Past/Never Smoking	Std rates Current/Past/Never Smoking	HR[1] (95% CI)		HR[2] (95% CI)	
			Current smoking	Past smoking	Current smoking	Past smoking
**RESPIRATORY SYSTEM**						
Chronic lung disease	125/219/60	1.83/0.40/0.06	**41.04 (29.90-56.35)**	**6.96 (5.20-9.32)**	**36.32 (26.18-50.4)**	**7.24 (5.37-9.76)**
Lower respiratory infections	17/82/133	0.39/0.15/0.13	**3.37 (2.00-5.65)**	1.28 (0.96-1.71)	**3.31 (1.95-5.61)**	**1.44 (1.07-1.95)**
Other diseases of respiratory system	26/159/173	0.38/0.27/0.17	**2.66 (1.75-4.05)**	**1.56 (1.25-1.95)**	**2.65 (1.72-4.06)**	**1.68 (1.33-2.12)**
**CIRCULATORY SYSTEM**						
Peripheral arterial disease	22/81/67	0.34/0.12/0.07	**5.83 (3.54-9.58)**	**2.07 (1.48-2.90)**	**4.80 (2.87-8.02)**	**2.00 (1.41-2.84)**
Coronary heart disease	143/572/809	1.84/0.92/0.79	**3.05 (2.54-3.66)**	**1.25 (1.11-1.39)**	**2.78 (2.30-3.36)**	**1.31 (1.17-1.47)**
Cerebrovascular disease	70/321/598	1.01/0.56/0.58	**2.43 (1.89-3.13)**	1.02 (0.89-1.18)	**2.30 (1.78-2.98)**	1.04 (0.90-1.20)
Other diseases of circulatory system	54/274/493	0.95/0.47/0.47	**2.19 (1.64-2.92)**	1.06 (0.91-1.24)	**2.04 (1.52-2.73)**	1.09 (0.93-1.28)
**NEOPLASMS**						
Cancer of lung	261/488/144	2.70/0.73/0.13	**21.98 (17.85-27.08)**	**5.75 (4.76-6.94)**	**17.85 (14.38-22.17)**	**5.52 (4.56-6.69)**
Oro-pharyngeal cancers	24/30/20	0.22/0.04/0.02	**11.02 (5.99-20.28)**	**2.13 (1.20-3.79)**	**7.86 (4.11-15.02)**	**1.89 (1.05-3.41)**
Unknown primary site	50/120/138	0.54/0.20/0.13	**5.17 (3.71-7.23)**	**1.53 (1.19-1.97)**	**4.42 (3.13-6.24)**	**1.45 (1.12-1.88)**
Cancer of oesophagus	27/82/64	0.20/0.11/0.06	**4.33 (2.74-6.86)**	**1.78 (1.27-2.48)**	**4.10 (2.55-6.59)**	**1.84 (1.31-2.59)**
Cancer of liver	33/82/89	0.26/0.12/0.09	**3.64 (2.42-5.47)**	1.33 (0.98-1.80)	**3.14 (2.05-4.80)**	1.32 (0.97-1.82)
Cancer of urinary tract	11/57/52	0.12/0.08/0.05	**3.04 (1.56-5.91)**	**1.73 (1.17-2.56)**	**2.96 (1.50-5.84)**	**1.88 (1.26-2.79)**
Cancer of pancreas	56/149/211	0.55/0.24/0.19	**3.09 (2.29-4.17)**	1.18 (0.95-1.46)	**2.72 (2.00-3.71)**	1.10 (0.88-1.37)
Cancer of kidney	11/37/47	0.09/0.06/0.04	**2.70 (1.38-5.28)**	1.19 (0.77-1.85)	**2.52 (1.26-5.02)**	1.21 (0.77-1.91)
Cancer of stomach	15/45/68	0.13/0.06/0.07	**2.30 (1.30-4.07)**	0.98 (0.67-1.45)	**2.04 (1.14-3.67)**	1.04 (0.70-1.54)
Cancer of prostate	19/110/103	0.46/0.32/0.29	**2.08 (1.27-3.42)**	1.08 (0.82-1.41)	**2.01 (1.21-3.34)**	1.14 (0.86-1.51)
Cancer of large intestine	42/180/250	0.60/0.29/0.24	**2.14 (1.54-2.99)**	1.20 (0.99-1.47)	**1.82 (1.29-2.57)**	1.19 (0.97-1.46)
Non-Hodgkin's lymphoma	12/63/98	0.14/0.10/0.09	1.51 (0.82-2.77)	1.00 (0.72-1.39)	1.49 (0.80-2.76)	1.06 (0.76-1.47)
Leukaemia	17/81/138	0.20/0.12/0.13	1.45 (0.87-2.42)	0.86 (0.65-1.14)	1.49 (0.89-2.52)	0.90 (0.68-1.20)
Cancer of ovary	10/28/99	0.11/0.10/0.14	1.46 (0.75-2.82)	0.75 (0.49-1.15)	1.33 (0.68-2.61)	0.79 (0.51-1.21)
Cancer of breast	12/57/124	0.22/0.21/0.17	1.26 (0.69-2.29)	1.20 (0.88-1.65)	1.14 (0.62-2.09)	1.21 (0.88-1.68)
Cancer of brain	15/85/116	0.10/0.11/0.10	1.06 (0.61-1.81)	1.11 (0.83-1.47)	1.12 (0.65-1.95)	1.17 (0.88-1.56)
Other neoplasms	70/408/590	0.65/0.56/0.55	1.47 (1.14-1.89)	1.10 (0.97-1.25)	**1.34 (1.04-1.73)**	1.09 (0.95-1.25)
**SELECTED OTHER CAUSES**						
Cirrhosis or alcoholic liver	26/38/28	0.17/0.06/0.03	**7.37 (4.28-12.69)**	**2.08 (1.27-3.41)**	**3.29 (1.84-5.88)**	1.40 (0.84-2.33)
External causes	64/221/347	0.61/0.38/0.36	**2.07 (1.57-2.72)**	1.07 (0.90-1.27)	**1.85 (1.40-2.46)**	1.09 (0.91-1.30)
Dementia incl Alzheimer's disease	32/278/531	0.74/0.49/0.51	**1.64 (1.14-2.35)**	1.06 (0.91-1.24)	**1.64 (1.14-2.36)**	1.11 (0.95-1.30)
**CAUSES NOT LISTED ABOVE**	160/777/1223	2.12/1.29/1.18	**2.13 (1.80-2.52)**	**1.14 (1.04-1.25)**	**1.80 (1.51-2.14)**	**1.18 (1.08-1.30)**
**ALL-CAUSE MORTALITY**	1459/5284/6865	17.50/8.47/6.56	**3.21 (3.03-3.40)**	**1.34 (1.29-1.39)**	**2.82 (2.66-3.00)**	**1.37 (1.32-1.43)**

Std rates: Age-sex standardised rates per 1000 person-years. HR[1]: adjusted for age as the underlying variable and sex (where applicable). HR[2]: additionally adjusted for region of residence, alcohol consumption, income, education, private health insurance and country of birth, with additional outcome-specific adjustments as outlined in Table S5 (as applied in Figure 1); bold font indicates statistical significance at $p<0.05$.

Table S8. Risk of death from selected causes by age and sex for current and past versus never smoking

Cause of death	Men					Women				
	Events	Rate*	HR	LCI	UCI	Events	Rate*	HR	LCI	UCI
**Chronic lung disease**										
**45-74 years**										
Never smoked	<5	<0.01				<5	<0.01			
Past smoking	22	0.02				19	0.03			
Current smoking	39	0.24				28	0.19			
**≥75 years**										
Never smoked	<20	0.02	1.00			<40	0.03	1.00		
Past smoking	113	0.13	4.77	3.45	6.60	65	0.22	6.46	4.81	8.68
Current smoking	34	0.64	21.90	14.76	32.50	24	0.76	22.36	15.17	32.96
**Coronary heart disease**										
**45-64 years**										
Never smoked	50	0.05	1.00			16	0.01	1.00		
Past smoking	32	0.05	0.95	0.72	1.25	12	0.02	1.46	0.93	2.32
Current smoking	44	0.29	2.81	2.12	3.74	17	0.10	4.64	2.97	7.26
**65-74 years**										
Never smoked	50	0.05	1.00			33	0.02	1.00		
Past smoking	66	0.06	1.24	0.98	1.58	13	0.02	1.06	0.74	1.51
Current smoking	22	0.13	1.48	1.01	2.18	12	0.10	2.66	1.66	4.25
**≥75 years**										
Never smoked	250	0.28	1.00			410	0.38	1.00		
Past smoking	325	0.36	1.42	1.26	1.60	124	0.42	1.24	1.07	1.43
Current smoking	31	0.55	1.94	1.46	2.57	17	0.67	1.95	1.43	2.66
**Cerebrovascular disease**										
**45-64 years**										
Never smoked	12	0.01	1.00			13	0.01	1.00		
Past smoking	5	0.01	0.68	0.39	1.16	10	0.01	1.37	0.82	2.29
Current smoking	10	0.06	2.58	1.53	4.35	11	0.05	3.47	2.05	5.88
**65-74 years**										
Never smoked	20	0.02	1.00			26	0.02	1.00		
Past smoking	26	0.03	1.32	0.95	1.83	11	0.02	0.83	0.55	1.25
Current smoking	10	0.07	1.95	1.17	3.24	8	0.06	2.27	1.33	3.85
**≥75 years**										
Never smoked	180	0.2	1.00			347	0.32	1.00		
Past smoking	184	0.2	1.03	0.89	1.20	85	0.29	0.96	0.81	1.14
Current smoking	18	0.33	1.68	1.18	2.39	13	0.45	1.28	0.85	1.92
**Cancer of lung**										
**45-64 years**										
Never smoked	10	0.01	1.00			25	0.02	1.00		
Past smoking	28	0.04	4.06	2.68	6.16	44	0.06	4.50	3.28	6.17
Current smoking	46	0.26	13.85	9.07	21.16	46	0.22	13.60	9.77	18.93
**65-74 years**										
Never smoked	15	0.01	1.00			13	0.01	1.00		
Past smoking	91	0.09	6.08	4.20	8.81	56	0.08	9.95	6.61	14.98
Current smoking	56	0.35	17.58	11.77	26.26	36	0.25	27.34	17.48	42.78
**≥75 years**										
Never smoked	33	0.04	1.00			48	0.04	1.00		
Past smoking	187	0.22	4.14	3.16	5.43	82	0.25	5.22	3.94	6.90
Current smoking	46	0.73	12.36	8.70	17.57	31	0.89	15.92	10.99	23.07

Table S8. Risk of death from selected causes by age and sex for current and past versus never smoking (continued)

Cause of death	Men					Women				
	Events	Rate*	HR	LCI	UCI	Events	Rate*	HR	LCI	UCI
**ALL-CAUSE MORTALITY**										
**45-64 years**										
Never smoked	385	0.44	1.00			381	0.26	1.00		
Past smoking	361	0.58	1.37	1.26	1.49	274	0.42	1.53	1.39	1.67
Current smoking	328	2.18	2.71	2.46	2.99	191	0.96	2.70	2.42	3.02
**65-74 years**										
Never smoked	506	0.48	1.00			567	0.34	1.00		
Past smoking	741	0.72	1.45	1.35	1.56	391	0.54	1.51	1.39	1.64
Current smoking	282	1.77	2.61	2.36	2.89	165	1.25	2.77	2.45	3.13
**≥75 years**										
Never smoked	1917	2.17	1.00			3109	2.87	1.00		
Past smoking	2474	2.77	1.28	1.22	1.34	1043	3.43	1.24	1.18	1.31
Current smoking	303	5.13	2.09	1.90	2.29	190	6.22	2.10	1.88	2.34

HRs are from fully adjusted models (as in Figure 1). HRs were not estimated for mortality from chronic lung disease in 45-64-year-olds due to small number of deaths. In analyses stratified by age group, each participant contributed their follow-up time to one or more age categories, splitting the follow-up time if participants moved to the next age group during follow-up.

*Crude rate per 1000 person years. Hazard ratios are adjusted for age, sex, region of residence, alcohol consumption, annual household income, education attainment, country of birth (Australia vs. Other) and private health insurance; cancer of lung was also adjusted for fruit intake.

Table S9. Sex specific HRs, where interaction between sex and smoking status was identified in main analysis

Cause of death	Men				Women				Total			
	Deaths	HR	LCI	UCI	Deaths	HR	LCI	UCI	Deaths	HR	LCI	UCI
**NEOPLASMS**												
**Cancer of oesophagus**												
Never smoked	32	1.00			32	-			64	1.00		
Past smoking	70	2.41	1.57	3.71	12	-	-	-	82	1.84	1.31	2.59
Current smoking	<27	6.42	3.73	11.07	<5	-	-	-	27	4.10	2.55	6.59
**Cancer of stomach**												
Never smoked	32	1.00			36	-			68	1.00		
Past smoking	39	1.45	0.89	2.34	6	-	-	-	45	1.04	0.70	1.54
Current smoking	<15	2.83	1.41	5.69	<5	-	-	-	15	2.04	1.14	3.67
**Cancer of liver**												
Never smoked	49	1.00			40	-			89	1.00		
Past smoking	61	1.31	0.89	1.92	21	-	-	-	82	1.32	0.97	1.82
Current smoking	<33	3.80	2.35	6.14	<5	-	-	-	33	3.14	2.05	4.80
**SELECTED OTHER CAUSES**												
**External causes**												
Never smoked	168	1.00			179	1.00			347	1.00		
Past smoking	144	1.02	0.81	1.28	77	1.31	0.99	1.72	221	1.09	0.91	1.30
Current smoking	49	2.01	1.44	2.82	15	1.38	0.80	2.39	64	1.85	1.40	2.46
**Dementia including Alzheimer's disease**												
Never smoked	164	1.00			367	1.00			531	1.00		
Past smoking	191	1.25	1.01	1.56	87	0.95	0.74	1.21	278	1.11	0.95	1.30
Current smoking	16	1.61	0.95	2.71	16	1.69	1.02	2.80	32	1.64	1.14	2.36

HRs were not estimated for mortality in females from cancer of oesophagus, stomach or liver due to small number of deaths

Table S10. Smoking patterns at resurvey by smoking status at recruitment

	Never smoking at recruitment	Ex-smoking at recruitment	Current smoking at recruitment, by cigarettes per day			
MEN			≤14	15-24	≥25	All amounts*
Current smoking at re-survey (%)	0.1%<br>34/25840	1.6%<br>313/19479	47.1%<br>503/1067	59.6%<br>714/1197	67.5%<br>626/928	57.2%<br>1864/3259
Cigarettes smoked per day at resurvey (SD)	7.2(7.8)	13.9(8.0)	9.0(4.8)	17.4(6.7)	26.2(10.0)	18.0(10.1)
WOMEN						
Current smoking at re-survey (%)	0.1%<br>61/42532	1.8%<br>331/18752	52.8%<br>769/1456	65.8%<br>996/1513	69.9%<br>510/730	61.0%<br>2300/3770
Cigarettes smoked per day at resurvey (SD)	8.3(6.9)	11.6(7.0)	9.3(4.8)	16.2(5.2)	24.1(7.9)	15.6(8.0)

Follow-up data on smoking status from Wave 2 (2012-2015) supplemented by that from the Social, Environmental and Economic Factors (SEEF) sub-study (2010). Of the study participants, 64,537 (36.2%) had missing follow up data on smoking. Among those with follow up data, 0.1% of those who never smoked, 1.7% of those who reported past smoking and 59.2% of those who reported current smoking at recruitment reported current smoking at follow-up.

SD: standard deviation.

*Numbers do not sum to total due to missing values.

Table S11. Hazard ratios for current versus never smoking: Sensitivity analysis, with additional adjustment for BMI

Cause of death	HR* (95% CI) as in Figure 1	HR* (95% CI) additionally adjusted for BMI
**RESPIRATORY SYSTEM**		
Chronic lung disease	36.32 (26.18-50.4)	32.82 (23.6-45.65)
Lower respiratory infections	3.31 (1.95-5.61)	3.15 (1.85-5.36)
Other diseases of respiratory system	2.65 (1.72-4.06)	2.59 (1.69-3.99)
**CIRCULATORY SYSTEM**		
Peripheral arterial disease	4.80 (2.87-8.02)	4.68 (2.80-7.85)
Coronary heart disease	2.78 (2.30-3.36)	2.77 (2.28-3.33)
Cerebrovascular disease	2.30 (1.78-2.98)	2.22 (1.71-2.87)
Other diseases of circulatory system	2.04 (1.52-2.73)	2.04 (1.52-2.74)
**NEOPLASMS**		
Cancer of lung	17.85 (14.38-22.17)	17.40 (14-21.63)
Oro-pharyngeal cancers	7.86 (4.11-15.02)	7.06 (3.66-13.6)
Unknown primary site	4.42 (3.13-6.24)	4.49 (3.18-6.35)
Cancer of oesophagus	4.10 (2.55-6.59)	3.94 (2.44-6.36)
Cancer of liver	3.14 (2.05-4.80)	3.14 (2.05-4.81)
Cancer of urinary tract	2.96 (1.50-5.84)	3.08 (1.56-6.08)
Cancer of pancreas	2.72 (2.00-3.71)	2.72 (1.99-3.71)
Cancer of kidney	2.52 (1.26-5.02)	2.52 (1.26-5.04)
Cancer of stomach	2.04 (1.14-3.67)	2.02 (1.12-3.63)
Cancer of prostate	2.01 (1.21-3.34)	2.12 (1.27-3.52)
Cancer of large intestine	1.82 (1.29-2.57)	1.85 (1.31-2.61)
Non-Hodgkin's lymphoma	1.49 (0.80-2.76)	1.48 (0.79-2.74)
Leukaemia	1.49 (0.89-2.52)	1.48 (0.88-2.50)
Cancer of ovary	1.33 (0.68-2.61)	1.35 (0.69-2.65)
Cancer of breast	1.14 (0.62-2.09)	1.18 (0.64-2.18)
Cancer of brain	1.12 (0.65-1.95)	1.11 (0.64-1.93)
Other neoplasms	1.34 (1.04-1.73)	1.34 (1.04-1.74)
**SELECTED OTHER CAUSES**		
Cirrhosis or alcoholic liver	3.29 (1.84-5.88)	3.66 (2.05-6.53)
External causes	1.85 (1.40-2.46)	1.53 (1.06-2.21)
Dementia incl Alzheimer’s disease	1.64 (1.14-2.36)	1.79 (1.35-2.38)
**CAUSES NOT LISTED ABOVE**	1.80 (1.51-2.14)	1.78 (1.48-2.15)
**ALL-CAUSE MORTALITY**	2.82 (2.66-3.00)	2.82 (2.66-3.00)

HR*: Hazard ratios adjusted for age as the underlying time variable, sex, region of residence, alcohol consumption, income, education, private health insurance and country of birth, with additional outcome-specific adjustments as outlined in Table S5.

Table S12. Hazard ratios for current and past versus never smoking: Sensitivity analysis, with additional adjustment for physical activity tertile

Cause of death	HR* (95% CI) as in Fig 1	HR* (95% CI) additionally adjusted for physical activity tertile
**RESPIRATORY SYSTEM**		
**Chronic lung disease**		
Never smoked	1	1
Past smoking	7.24 (5.37-9.76)	7.23 (5.37-9.73)
Current smoking	36.32 (26.18-50.4)	35.7 (25.75-49.48)
**CIRCULATORY SYSTEM**		
**Coronary heart disease**		
Never smoked	1	1
Past smoking	1.31 (1.17-1.47)	1.28 (1.14-1.44)
Current smoking	2.78 (2.30-3.36)	2.76 (2.29-3.33)
**Cerebrovascular disease**		
Never smoked		
Past smoking	1.04 (0.90-1.20)	1.05 (0.9-1.21)
Current smoking	2.30 (1.78-2.98)	2.28 (1.77-2.96)
**NEOPLASMS**		
**Cancer of lung**		
Never smoked	1	1
Past smoking	5.52 (4.56-6.69)	5.55 (4.58-6.72)
Current smoking	17.85 (14.38-22.17)	17.79 (14.33-22.1)

HR*: Hazard ratios adjusted for age as the underlying time variable, sex, region of residence, alcohol consumption, income, education, private health insurance and country of birth, with additional outcome-specific adjustments as outlined in Table S5.

Table S13. Deaths, death rates and hazard ratios for current and past versus never smoking: Sensitivity analysis broadening the definition of "current smoking" to include those who quit 3 years prior to baseline

Cause of death	Deaths	Std rate	HR	LCI	UCI
**RESPIRATORY SYSTEM**					
**Chronic lung disease**					
Never smoked	61	0.06	1.00		
Past smoking	192	0.35	6.42	4.75	8.67
Current smoking	152	1.85	37.07	26.98	50.94
**CIRCULATORY SYSTEM**					
**Coronary heart disease**					
Never smoked	809	0.79	1.00		
Past smoking	545	0.9	1.25	1.11	1.40
Current smoking	170	1.73	2.71	2.27	3.23
**Cerebrovascular disease**					
Never smoked	598	0.58	1.00		
Past smoking	312	0.56	1.03	0.89	1.20
Current smoking	79	0.94	2.14	1.67	2.73
**NEOPLASMS**					
**Cancer of lung**					
Never smoked	144	0.13	1.00		
Past smoking	422	0.67	4.98	4.10	6.06
Current smoking	327	2.67	18.44	14.95	22.73

Std rates: Age-sex standardised rates per 1000 person-years. Hazard ratios adjusted for age as the underlying time variable, sex, region of residence, alcohol consumption, income, education, private health insurance and country of birth, with additional outcome-specific adjustments as outlined in Table S5.
LCI: Lower confidence limit. UCI: Upper confidence limit.

Table S14. Deaths, death rates and hazard ratios for deaths from diseases of the respiratory system, with varying exclusions for respiratory diseases at baseline

Cause of death	Deaths		Std rate		HR	LCL	UCL
	Current Smoking	Never Smoked	Current Smoking	Never Smoked			
**Sensitivity analysis, excluding participants with cancer, CVD or disease specific hospitalisation***							
Chronic lung disease	125	61	1.83	0.06	35.81	25.85	49.60
Lower respiratory infections	17	135	0.38	0.13	3.18	1.87	5.39
Other diseases of respiratory system	28	176	0.38	0.18	2.74	1.81	4.14
**Sensitivity analysis, excluding participants with cancer or CVD (no exclusion for history of respiratory disease at baseline)**							
Chronic lung disease	149	75	2.02	0.07	32.43	24.18	43.49
Lower respiratory infections	17	137	0.37	0.13	3.04	1.80	5.15
Other diseases of respiratory system	30	202	0.39	0.19	2.60	1.75	3.87
**Main analysis, excluding participants with cancer or CVD or chronic lower respiratory diseases#**							
Chronic lung disease	125	60	1.83	0.06	36.32	26.18	50.40
Lower respiratory infections	17	133	0.39	0.13	3.31	1.95	5.61
Other diseases of respiratory system	26	173	0.38	0.17	2.65	1.72	4.06

**For each outcome [chronic lung disease (J40–44), lower respiratory infections (J09-J18, J20-J22, J85-J86) or other diseases of the respiratory system (J00-J99 other than J40-44, J09-J18, J20-J22, J85-J85)], participants with a history of hospitalisation for that outcome in the 5 years prior to baseline were excluded.*

# For each outcome, participants with a history of hospitalisation for chronic respiratory diseases in the 5 years prior to baseline (ICD-10 codes J40-J44, J47) were excluded; these codes do not include asthma.

Table S15. Deaths, death rates and hazard ratios for current versus never smoking: Sensitivity analysis using pack-years of smoking as the exposure

Cause of death	Deaths	Std rate	HR	LCI	UCI
**Chronic lung disease**					
Never smoked	60	0.06	1.00		
Current smoking					
Less than 20 pack-years	8	0.86	17.18	8.01	36.83
20 to <40 pack-years	34	1.29	31.84	20.31	49.90
≥40 pack-years	76	3.16	61.47	41.05	92.04
**Coronary heart disease**					
Never smoked	809	0.79	1.00		
Current smoking					
Less than 20 pack-years	17	1.12	1.99	1.22	3.24
20 to <40 pack-years	50	2.03	2.84	2.11	3.82
≥40 pack-years	70	2.53	3.66	2.81	4.76
**Cerebrovascular disease**					
Never smoked	598	0.58	1.00		
Current smoking					
Less than 20 pack-years	8	0.61	1.57	0.77	3.18
20 to <40 pack-years	21	0.96	2.02	1.29	3.14
≥40 pack-years	33	1.04	2.93	2.03	4.23
**Cancer of lung**					
Never smoked	144	0.13	1.00		
Current smoking					
Less than 20 pack-years	23	1.67	8.99	5.70	14.16
20 to <40 pack-years	69	2.12	15.39	11.32	20.92
≥40 pack-years	147	3.91	29.29	22.47	38.16

Pack-years were calculated by multiplying the number of years smoked by the number of packs smoked per day (the number of cigarettes smoked per day divided by 20, assuming that a pack contained 20 cigarettes). Pack-years were categorised as <20, 20 to 40 and ≥40, approximately based on tertiles of pack-years in current smokers.

Hazard ratios adjusted for age as the underlying time variable, sex, region of residence, alcohol consumption,income, education, private health insurance and country of birth, with additional outcome-specific adjustments as outlined in Table S5.

Table S16. Hazard ratios for current versus never smoking: Sensitivity analysis excluding heavy drinkers (≥15 drinks/week), for causes for which alcohol is a known risk factor

Cause of death	HR (95% CI) Main analysis as in Figure 1	HR (95% CI) Excluding heavy drinkers
**NEOPLASMS**		
Oro-pharyngeal cancers	7.86 (4.11-15.02)	6.18 (2.74-13.95)
Cancer of oesophagus	4.10 (2.55-6.59)	2.70 (1.46-5.02)
Cancer of liver	3.14 (2.05-4.80)	2.94 (1.78-4.85)
Cancer of large intestine	1.82 (1.29-2.57)	1.76 (1.18-2.62)
Cancer of breast	1.14 (0.62-2.09)	1.05 (0.54-2.04)
**SELECTED OTHER CAUSES**		
External causes	1.85 (1.40-2.46)	1.76 (1.28-2.42)
Cirrhosis or alcoholic liver	3.29 (1.84-5.88)	3.51 (1.50-8.23)

Hazard ratios adjusted for age as the underlying time variable, sex, region of residence, alcohol consumption, income, education, private health insurance and country of birth, with additional outcome-specific adjustments as outlined in Additional file 1: Table S5.

Table S17. Estimates of smoking attributable deaths in Australia, by age group and sex, 2019

Cause of death	Men			Women			Total		
	45-74y	≥75y	≥45y	45-74y	≥75y	≥45y	45-74y	≥75y	≥45y
Chronic lung disease	1175	2096	3271	968	1549	2517	2143	3645	5788
Lower respiratory infections	75	268	342	56	243	299	131	511	642
Other diseases of the respiratory system	162	400	562	82	214	295	243	614	857
Peripheral arterial disease	156	252	407	61	166	226	216	417	634
Coronary heart disease	948	1055	2003	250	458	708	1198	1513	2711
Cerebrovascular disease	146	138	284	114	118	231	259	256	515
Other diseases of the circulatory system	154	277	431	95	221	317	249	498	748
Cancer of lung	2066	1834	3900	1371	1005	2377	3437	2839	6276
Oro-pharyngeal cancers	307	148	455	71	13	84	378	161	539
Cancer of oesophagus	274	139	413	33	18	51	308	157	464
Cancer of liver	294	85	380	51	23	73	345	108	453
Cancer of the urinary tract	130	246	376	21	46	67	151	292	444
Cancer of pancreas	169	71	240	88	38	126	257	109	366
Cancer of kidney	71	34	105	12	9	22	83	44	127
Cancer of stomach	42	26	68	11	1	12	53	27	80
Cancer of prostate	119	237	356	-	-	-	119	237	356
Cancer of large intestine	165	117	282	66	71	137	231	188	693
Cancer of unknown primary site	254	190	444	120	129	274	374	319	427
Other neoplasms	143	149	292	81	55	150	223	204	482
Cirrhosis or alcoholic liver	294	24	318	136	27	160	430	51	591
External causes	309	141	449	77	65	154	386	205	673
Dementia incl Alzheimer's disease	39	334	374	26	273	305	65	607	2363
*Causes not included in the selected cause list**	*660*	*860*	*1520*	*328*	*516*	*871*	988	1375	*693*
Total number of deaths attributable to smoking	7493	8261	15753	3790	4742	8532	11283	13002	24285
Deaths attributable to current smoking	4480	2019	6499	2191	1518	3709	6671	3537	10208
Deaths attributable to past smoking	3013	6242	9255	1599	3223	4823	4612	9465	14077
Total number of all-cause deaths in the population	*29597*	*51757*	*81354*	*18730*	*58341*	*77071*	*48327*	*110098*	*158425*
% of deaths attributable to smoking	25.3%	16.0%	19.4%	20.2%	8.1%	11.1%	23.3%	11.8%	15.3%
% of deaths attributable to current smoking	15.1%	3.9%	8.0%	11.7%	2.6%	4.8%	13.8%	3.2%	6.4%
% of deaths attributable to past smoking	10.2%	12.1%	11.4%	8.5%	5.5%	6.3%	9.5%	8.6%	8.9%

**Not included in the total number of smoking attributable deaths. The number of attributable deaths were based on summation of age group and sex-specific attributable deaths. As current smoking was not associated in the study with significantly increased risk of death from non–Hodgkin's lymphoma, leukaemia or cancers of ovary, breast and brain, they were not included in the calculations.*

Table S18. Estimates of smoking-attributable fractions of death for current and past smoking, by age and sex

Cause of death	Current smoking						Past smoking					
	Male			Female			Male			Female		
	45-74y	≥75y	≥45y	45-74y	≥75y	≥45y	45-74y	≥75y	≥45y	45-74y	≥75y	≥45y
Chronic lung disease	0.6117	0.1992	0.3114	0.5446	0.1852	0.2873	0.3058	0.5867	0.5103	0.3275	0.4446	0.4114
Lower respiratory infections	0.1232	0.0427	0.0546	0.1163	0.0399	0.0477	0.1258	0.1403	0.1382	0.1273	0.0728	0.0783
Other diseases of the respiratory system	0.1074	0.0366	0.0561	0.0859	0.0158	0.0296	0.2303	0.2090	0.2148	0.1535	0.1235	0.1294
Peripheral arterial disease	0.1979	0.0558	0.0999	0.1827	0.0880	0.1063	0.2813	0.3028	0.2961	0.1923	0.1389	0.1493
Coronary heart disease	0.1601	0.0328	0.0714	0.1803	0.0198	0.0450	0.0878	0.1269	0.1151	0.0574	0.0533	0.0539
Cerebrovascular disease	0.1362	0.0266	0.0462	0.1359	0.0165	0.0345	0.0144	0.0185	0.0178	0.0102	0.0073	0.0078
Other diseases of the circulatory system	0.0707	0.0277	0.0387	0.0850	0.0195	0.0287	0.0368	0.0393	0.0387	0.0234	0.0162	0.0173
Cancer of lung	0.3914	0.1632	0.2729	0.3518	0.1818	0.2800	0.3961	0.5757	0.4894	0.3722	0.4170	0.3912
Oro-pharyngeal cancers	0.3782	0.2095	0.3015	0.3357	0.0000	0.2297	0.1727	0.2449	0.2055	0.1811	0.0785	0.1487
Cancer of oesophagus	0.2268	0.0652	0.1536	0.0445	0.0000	0.0168	0.2283	0.2755	0.2497	0.1880	0.0815	0.1217
Cancer of liver	0.2243	0.0550	0.1498	0.0440	0.0000	0.0216	0.1043	0.1056	0.1049	0.1017	0.0606	0.0808
Cancer of the urinary tract	0.1273	0.0390	0.0688	0.0946	0.0228	0.0462	0.2881	0.2845	0.2857	0.1003	0.1130	0.1089
Cancer of pancreas	0.1503	0.0492	0.1074	0.1022	0.0241	0.0620	0.0335	0.0465	0.0390	0.0294	0.0225	0.0258
Cancer of kidney	0.1340	0.0262	0.0920	0.0431	0.0274	0.0335	0.0820	0.0905	0.0853	0.0620	0.0237	0.0386
Cancer of stomach	0.0927	0.0523	0.0737	0.0612	0.0000	0.0340	0.0166	0.0197	0.0181	0.0062	0.0038	0.0051
Cancer of prostate	0.0921	0.0234	0.0412	-	-	-	0.0409	0.0643	0.0582	-	-	-
Cancer of large intestine	0.0731	0.0259	0.0469	0.0367	0.0298	0.0325	0.0762	0.0770	0.0766	0.0557	0.0352	0.0432
Cancer of unknown primary site	0.2664	0.0489	0.1339	0.1963	0.0546	0.1171	0.1119	0.1601	0.1412	0.1065	0.0949	0.1000
Other neoplasms	0.0397	0.0076	0.0204	0.0180	0.0054	0.0116	0.0336	0.0434	0.0395	0.0286	0.0137	0.0210
Cirrhosis or alcoholic liver	0.2233	0.0000	0.1940	0.2652	0.0696	0.2021	0.1240	0.1071	0.1218	0.1088	0.1143	0.1106
External causes	0.0910	0.0280	0.0624	0.0563	0.0056	0.0254	0.0272	0.0398	0.0329	0.0226	0.0240	0.0235
Dementia incl Alzheimer's disease	0.0450	0.0147	0.0168	0.0378	0.0116	0.0133	0.0495	0.0511	0.0510	0.0224	0.0181	0.0183
*Causes not included in the cause list**	*0.0807*	*0.0195*	*0.0388*	*0.0618*	*0.0151*	*0.0268*	*0.0659*	*0.0745*	*0.0718*	*0.0487*	*0.0334*	*0.0373*

As current smoking was not associated in the study with significantly increased risk of death from Non–Hodgkin's lymphoma, leukaemia, or cancers of ovary, breast, or brain, they were not included in the calculations.

Table S19. Estimates of smoking-attributable fractions using within-study versus population prevalence of smoking

	using prevalence of smoking in the study population				using population level prevalence of smoking			
**Cause of death**	**Male**		**Female**		**Male**		**Female**	
	45-74y	≥75y	45-74y	≥75y	45-74y	≥75y	45-74y	≥75y
Chronic lung disease	0.918	0.786	0.872	0.630	0.933	0.811	0.899	0.691
Lower respiratory infections	0.249	0.183	0.244	0.113	0.333	0.212	0.295	0.144
Other diseases of the respiratory system	0.338	0.246	0.239	0.139	0.397	0.285	0.286	0.172
Peripheral arterial disease	0.479	0.359	0.375	0.227	0.546	0.397	0.440	0.277
Coronary heart disease	0.248	0.160	0.238	0.073	0.331	0.179	0.303	0.093
Cerebrovascular disease	0.151	0.045	0.146	0.024	0.219	0.045	0.200	0.032
Other diseases of the circulatory system	0.107	0.067	0.108	0.036	0.154	0.072	0.148	0.047
Cancer of lung	0.788	0.739	0.724	0.599	0.840	0.763	0.775	0.656
Oro-pharyngeal cancers	0.551	0.454	0.517	0.078	0.644	0.464	0.587	0.100
Cancer of oesophagus	0.455	0.341	0.232	0.082	0.526	0.372	0.271	0.103
Cancer of liver	0.329	0.161	0.146	0.061	0.405	0.179	0.176	0.075
Cancer of the urinary tract	0.415	0.323	0.195	0.136	0.468	0.362	0.249	0.170
Cancer of pancreas	0.184	0.096	0.132	0.047	0.262	0.097	0.178	0.060
Cancer of kidney	0.216	0.117	0.105	0.051	0.280	0.131	0.133	0.068
Cancer of stomach	0.109	0.072	0.067	0.004	0.160	0.067	0.101	0.005
Cancer of prostate	0.133	0.088	-	-	0.195	0.097	-	-
Cancer of large intestine	0.149	0.103	0.092	0.065	0.195	0.116	0.116	0.083
Unknown primary site	0.378	0.209	0.303	0.150	0.478	0.234	0.372	0.184
Other neoplasms	0.073	0.051	0.047	0.019	0.101	0.059	0.059	0.025
Cirrhosis or alcoholic liver	0.347	0.107	0.374	0.184	0.426	0.139	0.434	0.218
External causes	0.118	0.068	0.079	0.030	0.175	0.072	0.109	0.037
Dementia incl Alzheimer's disease	0.095	0.066	0.060	0.030	0.127	0.074	0.083	0.039
Smoking attributable deaths by age and sex	**7493**	**8261**	**3790**	**4742**	**8927**	**8871**	**4297**	**5591**
% Smoking attributable deaths by age and sex	**25.3%**	**16.0%**	**20.2%**	**8.1%**	**30%**	**17%**	**23%**	**10%**
Total smoking attributable deaths aged ≥45y	**24,285 (15.3%)**				**27,686 (17.5%)**			

Main analysis is based on prevalence of exposure among cases in the study. Sensitivity analysis is incorporating population-based prevalence of smoking as outlined in Table S6. Total numbers of attributable deaths were based on summation of age group and sex-specific attributable deaths.
