# Supplementary material for: Relationship of tobacco smoking to cause-specific mortality: contemporary estimates from Australia
Source: BMC Med. 2025 Feb 25;23:115. doi: 10.1186/s12916-025-03883-9 (PMC11854379; doi:10.1186/s12916-025-03883-9)
Supplement: Supplementary file 2 — Additional file 2. Literature review. [file 12916_2025_3883_MOESM2_ESM.pdf]

Relationship of tobacco smoking to cause-specific mortality: Contemporary estimates from Australia

Additional File 2: Rapid review of evidence on smoking and cause-specific mortality

Background

Tobacco smoking is a leading avoidable cause of morbidity and mortality internationally. Studies from countries other than Australia have shown that the risk of premature mortality from a range of causes is increased in current smokers, compared to people who have never smoked. Estimates of the number of deaths annually from smoking for Australia are based on applying tobacco-attributable fractions of deaths from international studies to local data. Studies estimating numbers of deaths attributable to smoking in Australia are outlined in Additional File 1, Table S1.

Direct empirical evidence on the relationship of smoking to premature cause-specific mortality and the proportion of deaths attributable to smoking in Australia is lacking. To our knowledge, there are no contemporary estimates of the number of deaths annually in Australia attributable to smoking derived from local relative risk data.

Aim of main study

This study aims to quantify, using direct data from the Australian population, the relationship of current and past tobacco smoking to cause-specific mortality and estimate the number and proportion of deaths attributable to tobacco smoking.

Aim of supporting rapid review

This rapid literature search aims to briefly summarise the peer-reviewed published literature on smoking cause-specific mortality to provide context for the current study and to enable consideration of the study findings with the existing body of evidence. The review was completed by one reviewer (AY).

Methods

Search strategy

Two databases, PubMed and Medline, were searched January 11th 2022, for peer-reviewed published literature on smoking cause-specific mortality. In addition to database searching, reference lists of identified studies will be manually searched for other relevant articles.

Two additional updates occurred, one on April 3rd 2023 and the other July 30th 2024, for any articles published since the original search.

Search terms

PubMed

1. (smoking OR smoker OR tobacco OR cigarette) AND ("cause-specific mortality" OR "cause-specific deaths")

MedLine

1. (smoking or smoker or tobacco or cigarette).mp
2. ("cause-specific mortality" or "cause-specific deaths").mp.
3. 1 and 2

Screening and data extraction

Publications identified via the database search were exported to Covidence, duplicates removed, and studies screened against eligibility criteria. A single reviewer completed all screening. Data from included studies was extracted into a predefined template.

Eligibility criteria

Population	Current adult combustible tobacco smokers<br>Studies in specific subpopulations were excluded (for example, patients with rheumatoid arthritis, HIV, or diabetes)
Intervention	Current smoking, no restriction on smoking intensity (cigarettes per day [CPD]) or frequency
Comparison	Adult never-smoker
Outcome	Any cause-specific mortality including but not limited to cancer, CVD and respiratory system
Outcome measures	Hazard ratio, risk ratio and population attributable fraction<br>(Note, prevalence of smoking cause-specific mortality not included)
Study design	Cohort studies<br>- No restriction on follow-up duration<br>- Nationally representative ideal (non-nationally representative studies will be considered where applicable)<br>Pooled meta-analyses of cohort studies
Setting	High-income countries
Date	None
Language	English
Other	Articles were excluded where full text was unavailable

Synthesis of study results

Results were narratively summarised with examples provided under specific health outcomes. Studies published prior to 2000 were included in the review but not summarised as they were considered less informative. Findings were used to inform the introduction and discussion of the main manuscript.

Results

Of the 771 studies identified via the database search, 38 articles were included (5, 6, 15-50: see main manuscript for references). The reference list of these was searched for additional publications with five new studies located (51-55).

Three studies were published prior to 2000 and are not discussed further (34, 38, 55).

Figure 1. PRIMA from database search

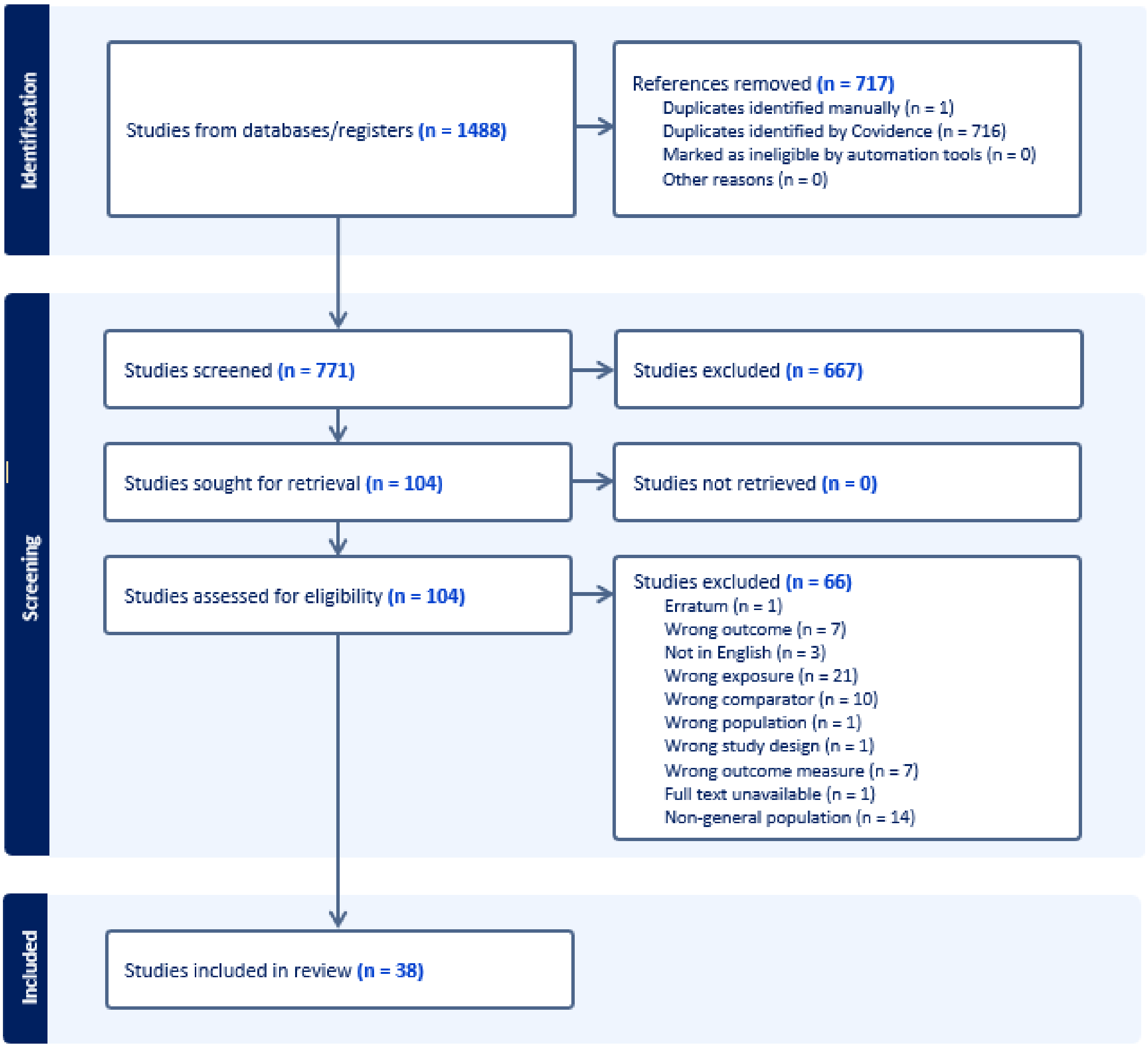

Summary of findings

- Overall, 43 publications were identified:
    - There were five pooled analyses of cohort studies from the Asia region (5, 6, 46, 47, 49). Meta-analysis included between 9 (5) and 34 (6) cohort studies.
    - There were 22 studies in which the population was nationally representative (17, 19, 20, 22, 24-26, 30, 33-36, 38, 39, 42, 43, 45, 50-54) and 16 non-nationally representative. (15, 16, 18, 21, 23, 27-29, 31, 32, 37, 40, 41, 44, 48, 55)
- The majority of studies were from the US, (5, 17-20, 24-29, 33, 35, 39, 43, 52, 53) Japan, (15, 37, 50) and Mexico. (21, 23, 45) Other countries also included Hong Kong, (31, 32) China, (42, 51) Estonia, (30) Lithuania, (36) Norway, (22) the Netherlands, (41) Thailand, (48) Italy, (40) Finland, (54) Cuba, (44) and UK. (16)
- Some studies included participants from 18 years and above (17, 22, 24, 26, 30, 35, 39, 53) while others included age restrictions (20+ years, (15, 38, 41) 25+ years, (23, 43) 30+ years, (20, 27, 28, 37, 43) 35+ years, (19, 21, 33, 36, 45, 51) 40+ years, (42, 50) 50+ years, (25, 29, 40, 52) 65+ years. (32)
- Follow-up varied from approximately 6-20 years.
- Several studies included only one sex (18, 22, 23, 27-29, 42, 54) but the majority included both sexes.

Cancer outcomes

- 37 studies reported a least one cancer outcome. (5, 6, 15-19, 21-33, 35-37, 39-41, 43-53)
- All cancer, smoking-related cancer and lung cancer were the most common cancer outcomes presented. Other cancers also reported included oral cavity, oesophagus, stomach, liver, colon/rectal/anus, pancreas, bladder, acute myeloid leukemia, prostate and ovarian.
- Almost all studies reported significantly greater cancer mortality risk for current smokers than never smokers. (6, 16-19, 22, 26-33, 36, 37, 41, 43, 45, 47, 49)
    - However, some studies found no difference in risk depending on the type of cancer (26, 41, 45) or sex. (15, 36)
- Former smokers were generally at a heightened risk of cancer than never smokers albeit less than current smokers. (15, 17, 19, 22, 26-28, 30-33, 36, 37, 39, 41, 43, 45)
    - Example from Gram et al: (22)
        - Total cancer: Former RR 1.17 (95% CI 1.03-1.33) compared to current RR 1.89 (95% CI 1.68-2.13).
        - Lung cancer: Former RR 2.40 (95% CI 1.43-4.03) compared to current RR 12.16 (95% CI 7.80-2.19.01).
        - Other smoking-related cancers: Former RR 1.24 (95% CI 1.03-1.51) compared to current RR 1.81 (95% CI 1.50-2.17).
    - No difference in risk was reported in some studies depending on the type of cancer (26, 33, 41, 45) or sex. (15, 36)
- Evidence consistently demonstrated a dose-response by CPD. (5, 15, 23, 25, 27, 31, 33, 37, 39, 45, 46, 52)
    - Example from Yang et al. (pooled cohort) for lung cancer, by CPD: (46)
        - <5 CPD HR: 2.0 (95% CI: 1.69-2.87) compared to ≥30 CPD HR: 8.69 (7.11-10.6).
    - Example from Qin et al. for all cancers, by CPD: (39)
        - 1-2 CPD HR: 2.28 (95% CI: 1.84-2.84) compared to >30 CPD HR: 5.26 (95% CI 4.54-6.10).
    - Some studies did not find there was a significant difference in risk depending on the type of cancer. (27, 33, 45)
- Evidence was suggestive of does response by smoking frequency. However, the relationship was not present across all studies and cancer types. (19, 21, 24, 53)
    - Example from Christensen et al of different cancers by smoking intensity: (19)
        - Smoking-related cancer: non-daily HR: 2.31 (95% CI: 2.01-2.65); daily HR: 4.33 (95% CI: 4.09-4.58).
        - Lung cancer: non-daily HR: 6.24 (95% CI: 5.17-7.54); daily HR: 12.74 (95% CI: 11.55-14.05).
        - Oral cancer: non-daily HR: 4.62 (95% CI: 1.84-11.58); daily HR: 9.74 (95% CI: 6.20-15.30).
    - Example from Inoue-Choi et al. for all cancer by smoking intensity: (24)
        - Daily HR: 3.14 (95% CI: 2.98-3.32); non-daily (previously daily) HR: 2.46 (95% CI: 2.14-2.83); and non-daily (lifelong) HR: 1.76 (95% CI: 1.41-2.21).
- Lung cancer generally had the highest HR compared to other cancer groups and also followed the same dose-response. (5, 18, 19, 22, 25-28, 30, 33, 41)
    - Example from Inoue-Choi et al. 2019, by current vs never smokers: (53)
        - Lung HR: 15.49 (95% CI: 12.64-18.99); oral HR: 5.32 (95% CI: 2.95-9.58); smoking-related HR: 4.49 (95% CI: 4.41-5.53); bladder HR: 4.48 (95% CI: 2.57-7.80); oesophagus HR: 3.26

(95% CI: 1.68-6.34); all HR: 2.79 (95% CI: 2.57 -3.04); pancreas HR: 2.29 (95% CI: 1.69-3.12); colon HR: 1.68 (95% CI: 1.30-2.19); stomach HR: 1.57 (95% CI: 0.92-2.68).
  - Example from Christensen et al., by smoking intensity: (19)
    - Lung cancer adjHR: former 4.15 (95% CI: 3.75-4.59); current 11.82 (95% CI: 10.73-13.03); non-daily 6.24 (95% CI: 5.17-7.54); Daily 12.74 (95% CI: 11.55-14.05).
  - Example from Inoue-Choi et al. 2017, by CPD: (25)
    - <1 CPD HR: 10.73 (7.59-15.15) compared to >30 CPD HR: 36.83 (95% CI 30.61-44.33).
- Evidence was suggestive that cancer mortality was associated with earlier age of smoking initiation and longer smoking duration. (35, 44, 46, 50)
  - Example from pooled cohort evidence from Yang et al, lung cancer mortality risk: (46)
    - Younger Age of initiation was associated with higher odds of lung cancer. HR current ranged from 6.32 (95% CI 5.02-7.94) for less than 20 years old to 3.5 (95% CI 1.90-55.2) for 45 or more years old.
    - Longer smoking duration was associated with higher odds of lung cancer, even at low CPD (<10 CPD). Among those who smoked 5-9 CPD, HR ranged from 2.57 (95% CI 1.33-4.98) for 15-24 years of smoking duration to 4.35 (95% CI 3.38-5.59) for 45 or more years.
  - Example from Liu et al., childhood smoking initiation and cancer risk: (35)
    - 6-9 years HR: 3.72 (2.97-4.65) compared to ≥18 years HR: 2.66 (95% CI 2.48-2.85).
- Population Attributable Fraction (PAF) was calculated in three studies.
  - Example from Gram et al: (22)
    - PAF total cancer: 24% (95% CI 18-31); PAF lung cancer: 79% (95% CI 72-86); PAF other smoking-related cancers: 24% (95% CI 14-34).
  - Example from Akter et al: (15)
    - PAF All cancer: Former 9.1%, current 10.3%; PAF smoking-related cancers: former 14.5%, current 18.0%.

Cardiovascular outcomes

- 38 studies reported at least one CVD outcome. (5, 6, 15-33, 35-37, 39-46, 48-51, 53, 54)
- CVD and cerebrovascular outcomes were the most common cardiovascular outcomes presented. Other related outcomes included: stroke, myocardial infarction, other vascular disease, (coronary and ischemic) heart disease, cerebrovascular, atherosclerosis, aortic aneurysm, other arterial disease, hypertensive heart disease, and circulatory disease.
- Compared to never smokers, current smokers had greater CVD mortality risk. (6, 17-19, 22, 26-33, 36, 37, 39, 42, 43, 47, 48)
  - Example from the pooled cohort study Yang et al. 2019, CVD mortality by gender: (47)
    - Men HR: 1.35 (95% CI 1.26-1.45). Women HR: 1.54 (95% CI 1.36-1.73)
  - Example from Tan et al. by CVD outcomes: (42)
    - CVD HR: 1.13 (95% CI 1.10-1.17); IHD HR: 1.19 (1.10-1.29); stroke HR: 1.13 (95% CI 1.08-1.18).
  - Some studies found no difference between current and never smokers (15, 41) or was dependent on the CVD outcome (33) or sex. (31, 36)
- Former smokers were generally found to have greater CVD mortality risk than never smokers. (17, 19, 26, 43, 45), (22, 27, 28, 30, 32, 33, 36, 42)
  - Example from Kenfield et al. 2008, by CVD outcomes: (27)
    - Total vascular disease HR: 1.32 (95% CI 1.20-1.44); CHD HR: 1.24 (1.09-1.42); cerebrovascular disease HR: 1.27 (95% CI 1.06-1.51).
  - However, some studies found no difference between former and never smokers (15) (21, 31, 37, 41, 48) or were dependent on the CVD outcome (19, 22, 26, 28, 30, 33, 45) or sex. (36)
- Evidence was suggestive that was a dose-response for CVD, heart disease and cerebrovascular disease, such that risk increased with greater CPD. (5, 15, 21, 23, 25, 27, 31, 33, 37, 39, 42, 45, 46)
  - Example from the pooled cohort study by Inoue-Choi et al. 2021, for Ischemic heart disease by CPD: (5)
    - Women: 1-2 CPD HR 2.24 (1.63-3.08) compared to >31 CPD HR: 10.64 (95% CI 5.00-22.66).
  - Example from Qin et al., cardiovascular mortality risk by CPD: (39)
    - CVD mortality: 1-2 CPD HR: 1.93 (95% CI: 1.53-2.36) compared to >30 CPD HR: 3.46 (95% CI: 2.89-4.15).
    - Heart disease: 1-2 CPD HR: 1.83 (95% CI: 1.43-2.33) compared to >30 CPD HR: 3.78 (95% CI: 3.12-4.58).

        - Cerebrovascular disease: 1-2 CPD HR: 2.49 (95% CI: 1.64-3.80) compared to >30 CPD HR: 2.35 (95% CI: 1.44-3.81).
    - Some studies found no difference in CVD mortality outcomes at lower CPD for some outcomes. (5, 15, 23, 31, 37)
- Evidence was varied in terms of the relationship between daily smoking and cardiovascular outcomes. Daily smokers were generally at a higher risk than non-daily smokers. (19, 21, 24, 26, 45, 53)
    - Example from Christensen et al., different CVD outcomes by smoking intensity: (19)
        - Circulatory – non-daily HR: 1.43 (95% CI: 1.30-1.57) vs daily HR: 1.63 (95% CI 1.57-1.70).
        - CVD – non-daily HR: 1 24 (95% CI: 1.11-1.39) vs daily HR: 1.47 (95% CI 1.40-1.54).
        - Cerebrovascular disease – non-daily HR: 1.39 (95% CI: 1.12-1.74) vs daily HR: 1.21 (95% CI 1.09 -1.35).
    - Some studies found this effect was dependent on the CVD outcome. (24)
- Evidence was suggestive that CVD risk increased with younger at initiation. (35, 44, 46, 50)
    - Example from the pooled cohort study Yang et al: (46)
        - Current CVD at < 20 years was HR1.74 (95% CI: 1.53-1.97) compared to HR: 1.27 (95% CI:1.12-1.43) the 35 or more years.
- Evidence was suggestive that CVD mortality risk remained relatively stable across smoking duration. (42, 46, 50)
    - Example from the pooled cohort study Yang et al: (46)
        - Current low intensity (5-9 CPD) CVD – HR: 1.38 (95% CI: 1.03-1.86) for less than 15 years compared to HR: 1.28 (95% CI: 1.13-1.44).
- Population Attributable Fraction (PAF) was calculated in two studies.
    - Example from Gram et al: (22)
        - PAF total CVD: 50% (95% CI 40-59); PAF circulatory disease: 63% (95% CI 49-77); PAF MI: 43% (95% CI 21-65); PAF cerebrovascular: 42% (95% CI 25-58).
    - Example from Akter et al: (15)
        - PAF CVD current: 21.7%.

Respiratory outcomes

- 26 studies reported at least one respiratory outcome. (5, 6, 18, 19, 21, 22, 24-28, 30-33, 35, 37, 39, 41, 43-46, 49, 51, 53)
- Respiratory disease and Chronic Obstructive Pulmonary Disease (COPD) were the most common respiratory outcomes presented. Other outcomes included other respiratory disease and chronic lower respiratory disease.
- Compared to never smokers, current smokers had high risk of respiratory disease mortality. (6, 18, 19, 22, 24, 26-28, 30, 32, 33, 37, 43, 45, 46, 49, 53)
    - Example from Cao et al:
        - Pulmonary disease HR: 8.27 (95% CI 5.76-11.86). (18)
    - Some studies reported no difference between current and never smokers, or was dependent on respiratory outcome (32, 45) or sex. (6, 32, 37)
- Former smokers were generally at a heightened risk of respiratory disease mortality than never smokers albeit less than current smokers (19, 22, 26-28, 30, 32, 33, 37, 39, 41, 43, 45, 46)
    - Example from Inoue-Choi et al. 2019: (53)
        - Chronic lower respiratory disease – former HR: 4.41 (95% CI 3.62-5.37) vs current HR: 12.24 (95% CI: 10.1-14.96).
    - There was no difference between former and never smokers in some studies, (21) or was dependent on respiratory disease outcome (45) or sex. (30, 37)
- There was a dose-response such that as CPD increased so did respiratory disease mortality risk. (5, 21, 25, 27, 31, 37, 39, 46)
    - Example from the pooled cohort study by Inoue-Choi et al. 2021: (5)
        - Men: 1-5 CPD HR: 2.69 (95% CI 1.66-4.37) compared to >40 CPD HR 7.15 (95% CI 3.90-13.12)
        - Women: 1-5 CPD HR: 5.29 (95% CI 2.33-12.04) compared to >11 CPD HR 6.83 (95% CI 4.19-11.12).
    - Example from Qin et al: (39)
        - Respiratory disease – 1-2 CPD HR: 9.75 (95% CI 6.15-15.46) compared to >30 CPD HR: 30.52 (95% CI: 22.65-41.15).
- Evidence was suggestive that respiratory disease mortality risk increased with increased smoking frequency. (19, 21, 24, 26, 45, 53)

    - Example from Christensen et al. by respiratory outcomes: (19)
        - Respiratory – Non-Daily HR: 4.19 (95% CI 3.51-5.00) vs daily HR: 5.77 (95% CI: 5.29-6.29).
        - COPD – Non-Daily HR: 7.66 (95% CI 6.09-9.64) vs daily HR: 11.62 (95% CI: 10.24-13.18).
    - However, this differed by outcome (45) in one study and another found no difference for non-daily use. (21)
- Evidence was suggestive that respiratory disease mortality risk increased with younger age at initiation (35, 44, 46)
    - Example from the pooled cohort study by Yang et al: (46)
        - Among 5-9 CPD smokers, initiating smoking at <20 years had a greater risk (HR 2.15; 95% CI: 1.69-2.73) compared to ≥35 years (HR: 1.22; 95% CI:0.86-1.71).
- Evidence was suggestive that respiratory disease mortality risk increased with greater smoking duration. (46)
    - Example from the pooled cohort study by Yang et al: (46)
        - Among those that smoked 5-9 CPD, <15 years HR: 1.67 (95% CI: 0.92-3.06) compared to ≥45 years HR: 1.81 (95% CI: 1.52-2.16).
- Population Attributable Fraction (PAF) was calculated in one study.
    - Example from Gram et al: (22)
        - PAF total respiratory disease: 76% (95% CI 63-88); PAF COPD: 85% (95% CI: 73-97); PAF other respiratory disease: 61% (34-88).

Other outcomes reported

- Other outcomes were reported in nine studies.
- Christensen et al. 2018 reported the HR for <u>diabetes mortality</u>- former HR: 1.18 (95% CI 1.05-1.33); current HR: 1.18 (95% CI 1.03-1.35); current non-daily HR: 1.14 (95% CI 0.82-1.57); current daily HR: 1.19 (95% CI 1.03-1.37). (19)
- Several other outcomes were reported by Thompson et al. 2021. These included <u>gastrointestinal</u> (Daily HR: 1.63; 95% CI 1.37-1.95); <u>renal</u> (Daily HR: 1.13; 95% CI 0.96-1.34); <u>hepatobiliary</u> (Daily HR: 1.13; 95% CI 0.99-1.30); <u>acute diabetic</u> (daily HR 1.03; 95% CI 0.73-1.52). (45)
- Lariscy et al. 2018 reported the RR (95% CI not provided) for current smokers for <u>intestinal ischemia</u> (female: 2.86; male 3.37; both p < .001); <u>liver cirrhosis</u> (female: 2.07; male: 3.64; both p < .001); <u>other digestive diseases</u> (female: 2.16; male: 2.18; both p < .001); <u>renal failure</u> (female: 1.40; male: 1.31; female p <0.05 and male not statistically significant). (33)
- Mortality from injuries, unknown, other and external causes was also calculated in five studies. (6, 19, 28, 30, 33, 44, 48, 53)
- Other analyses:
    - Thompson et al. 2022 also examined cause-specific mortality by race, sex and ethnicity (results for all participants reported above). (43)
    - Chen et al. examined cause-specific mortality by rurality and compared two separate cohort studies. (51)
